# Impact of ionizing radiation on cell-ECM mechanical crosstalk in breast cancer

**DOI:** 10.3389/fbioe.2024.1408789

**Published:** 2024-06-06

**Authors:** Rocco Mottareale, Crescenzo Frascogna, Giuseppe La Verde, Cecilia Arrichiello, Paolo Muto, Paolo A. Netti, Sabato Fusco, Valeria Panzetta, Mariagabriella Pugliese

**Affiliations:** ^1^ Department of Physics “E. Pancini”, University of Naples Federico II, Naples, Italy; ^2^ Institute of Applied Sciences and Intelligent Systems E. Caianiello (CNR-ISASI), Pozzuoli, Italy; ^3^ Center for Advanced Biomaterials for Healthcare @CRIB, Italian Institute of Technology, Naples, Italy; ^4^ Department of Chemical, Materials and Production Engineering, University of Naples Federico II, Naples, Italy; ^5^ Radiotherapy Unit, Istituto Nazionale Tumori-IRCCS-Fondazione “G. Pascale”, Naples, Italy; ^6^ Interdisciplinary Research Centre on Biomaterials (CRIB), University of Naples Federico II, Naples, Italy; ^7^ Department of Medicine and Health Sciences “V. Tiberio”, University of Molise, Campobasso, Italy

**Keywords:** breast adenocarcinoma, cytoskeleton, extracellular matrix, mechanotransduction, mechanobiology, radiotherapy

## Abstract

The stiffness of the extracellular matrix plays a crucial role in cell motility and spreading, influencing cell morphology through cytoskeleton organization and transmembrane proteins’ expression. In this context, mechanical characterization of both cells and the extracellular matrix gains prominence for enhanced diagnostics and clinical decision-making. Here, we investigate the combined effect of mechanotransduction and ionizing radiations on altering cells’ mechanical properties, analysing mammary cell lines (MCF10A and MDA-MB-231) after X-ray radiotherapy (2 and 10 Gy). We found that ionizing radiations sensitively affect adenocarcinoma cells cultured on substrates mimicking cancerous tissue stiffness (15 kPa), inducing an increased structuration of paxillin-rich focal adhesions and cytoskeleton: this process translates in the augmentation of tension at the actin filaments level, causing cellular stiffness and consequently affecting cytoplasmatic/nuclear morphologies. Deeper exploration of the intricate interplay between mechanical factors and radiation should provide novel strategies to orient clinical outcomes.

## 1 Introduction

The extracellular matrix (ECM) is a highly dynamic and ordered network of biochemically distinct components (glycoproteins, proteoglycans, polysaccharides and fibrous proteins) providing structural support and regulating cell signalling/communication (Nallanthighal et al., 2019). Increasing evidence suggests how the interplay between tumor cells and their microenvironment drives the initiation, progression, invasion, and metastasis of solid tumors (Levental et al., 2009; Nallanthighal et al., 2019). Within this dynamic process, the tumor microenvironment (TME) disrupts the normal cellular and matrix architecture through both biochemical and mechanical mechanisms, promoting s and resistance to treatment (Balachandra et al., 2022). The ECM undergoes modifications in content and distribution orchestrated by tumor cells. An imbalance in matrix production and degradation, particularly through excessive deposition and cross-linking of ECM proteins, is a primary contributor to pathological matrix stiffening (Stowers et al., 2017; Najafi et al., 2019; Panciera et al., 2020). The varying stiffness of the tumor ECM is evident across different stages of tumor progression, resulting from alterations in gene expression, signal transduction, and receptor-ligand interactions (Stowers et al., 2017; Najafi et al., 2019; Panciera et al., 2020). In the context of a bidirectional communication between ECM and the intracellular environment, a pivotal role is represented by the assembling of focal adhesions (FAs). FAs stand as specialized sites of adhesion developed by the co-localization of disparate complex of proteins such as integrins, talin and paxillin (Burridge and Chrzanowska-Wodnicka, 1996). FAs are primarily involved in mechanosensing, allowing cells to sense and respond to the mechanical properties of their microenvironment, through a process known as mechanotransduction (Fletcher and Mullins, 2010; Goldmann, 2012; Jansen et al., 2015). In this regard, FAs act as functional units mediating the connection between the ECM and the intracellular cytoskeleton (CSK) (Burridge and Chrzanowska-Wodnicka, 1996; Petit and Thiery, 2000). The CSK occurs as an intricate three-dimensional network of protein filaments within eukaryotic cells’ cytoplasm and serves as a dynamic scaffold operating crucial functions for cellular integrity and functionality (Hohmann and Dehghani, 2019) and for maintaining cellular mechanical homeostasis (Schliwa and Van Blerkom, 1981; Gefen and Weihs, 2016). In fact, beyond providing structural support, the CSK affects dynamic cellular processes, including cell adhesion, motility, and migration, in response to external cues (Pollard and Borisy, 2003; Sanghvi-Shah and Weber, 2017). Proceeding more inward onto mechanosensing machinery, integrins, trans-membrane proteins within FAs, act as receptors that physically link the ECM to the cell CSK (Fox et al., 1994; Pollard and Borisy, 2003; Sanghvi-Shah and Weber, 2017), enabling the propagation of mechanical signals to the cell interior up to the nucleus. In this context, CSK filaments intricately connect with the nucleus through the linker of nucleoskeleton and CSK (LINC) complex (Wang and Chen, 2013; De Pascalis and Etienne-Manneville, 2017). Moreover, nesprins, proteins positioned on the outer nuclear membrane, establish a physical link with actin filaments (Houben et al., 2007; Lombardi et al., 2011). Simultaneously, they create associations with the inner nuclear membrane through dimers of SUN-domain proteins, which, in turn, are linked to the nuclear lamina and chromatin (Lombardi et al., 2011). This physical interconnection facilitates the transmission of mechanical signals, influencing nuclear morphology (Chen et al., 2015) and affecting the organization of chromatin and the accessibility of the genome to the transcription machinery within the nucleus (Buxboim et al., 2010). Therefore, these alterations trigger a cascade of events that impact gene expression, ultimately influencing cell morphology and relative functions. At this point, considering the broad involvement of the CSK in cell physiology, it is unsurprising that anomalies in cytoskeletal proteins have been linked to several diseases, such as: cardiovascular diseases (Hein, 2000), neurodegenerative disorders (Benitez-King et al., 2004; Bamburg and Bloom, 2009), and cancer (Hall, 2009; Panzetta et al., 2017d). In the context of cancer, disruptions in cytoskeletal dynamics play a pivotal role in instigating hallmark characteristics such as uncontrolled proliferation (Provenzano and Keely, 2011), resistance to cell death (Groth-Pedersen et al., 2012; Al Absi et al., 2018) and the acquisition of metastatic potential (Yamaguchi and Condeelis, 2007). At the same time, the aforementioned stiff pathological ECM establishes a feedback loop fostering malignancies in tumor cells (Stowers et al., 2017). Cancer therapies, including radiotherapy (RT), exert profound effects on cellular structures, including the CSK (Gabryś et al., 2007; Panzetta et al., 2015; Panzetta et al., 2017a; Panzetta et al., 2017c; Panzetta et al., 2020; La Verde et al., 2021). In this respect, ionizing radiation (IR) is commonly used in clinical radiotherapy to eradicate the malignant population while sparing the normal tissue. RT puts its radiobiological bases into the accumulation of radiation-induced DNA damage into the target tumor population. More recently, research on radiation-induced effects on cells has shifted to mechanobiology: nowadays the evolving interest extends beyond the survival of cancer cells to investigate the role of IR in modifying the physical interactions between cells and their surrounding microenvironment (La Verde et al., 2021). Studies have highlighted how cells’ mechanical alterations are closely related to exposition doses, post-irradiation time and ECM stiffness depending on the specific cellular phenotype (La Verde et al., 2021; Frascogna et al., 2024). Tumor cells actively perceive and react to the mechanical cues in the TME, including strength, direction, and duration. In the case of breast adenocarcinoma, the aggressive triple-negative MDA-MB-231 cancer cell line and their normal epithelial counterpart MCF10A, have exhibited a dose-dependent increase in adhesion when cultured on a substrate mimicking the mechanical rigidity of tumor microenvironment (Panzetta et al., 2020; Frascogna et al., 2024). On the other hand, on physiological microenvironment both cell lines have exhibited a notable decrease in migration velocity, attributable to the existence of a potential radioprotective role related to the softer ECM (Panzetta et al., 2015; Panzetta et al., 2017c; Panzetta et al., 2019; Frascogna et al., 2024). In this last described case, only normal cells have exhibited a reduction of adhesion area proportional to the radiation absorbed dose, with MDA-MB-231 cells instead characterized by a dose dependent increase in adhesion area on the same physiological microenvironment (Panzetta et al., 2019; Frascogna et al., 2024).

**FIGURE 1:**
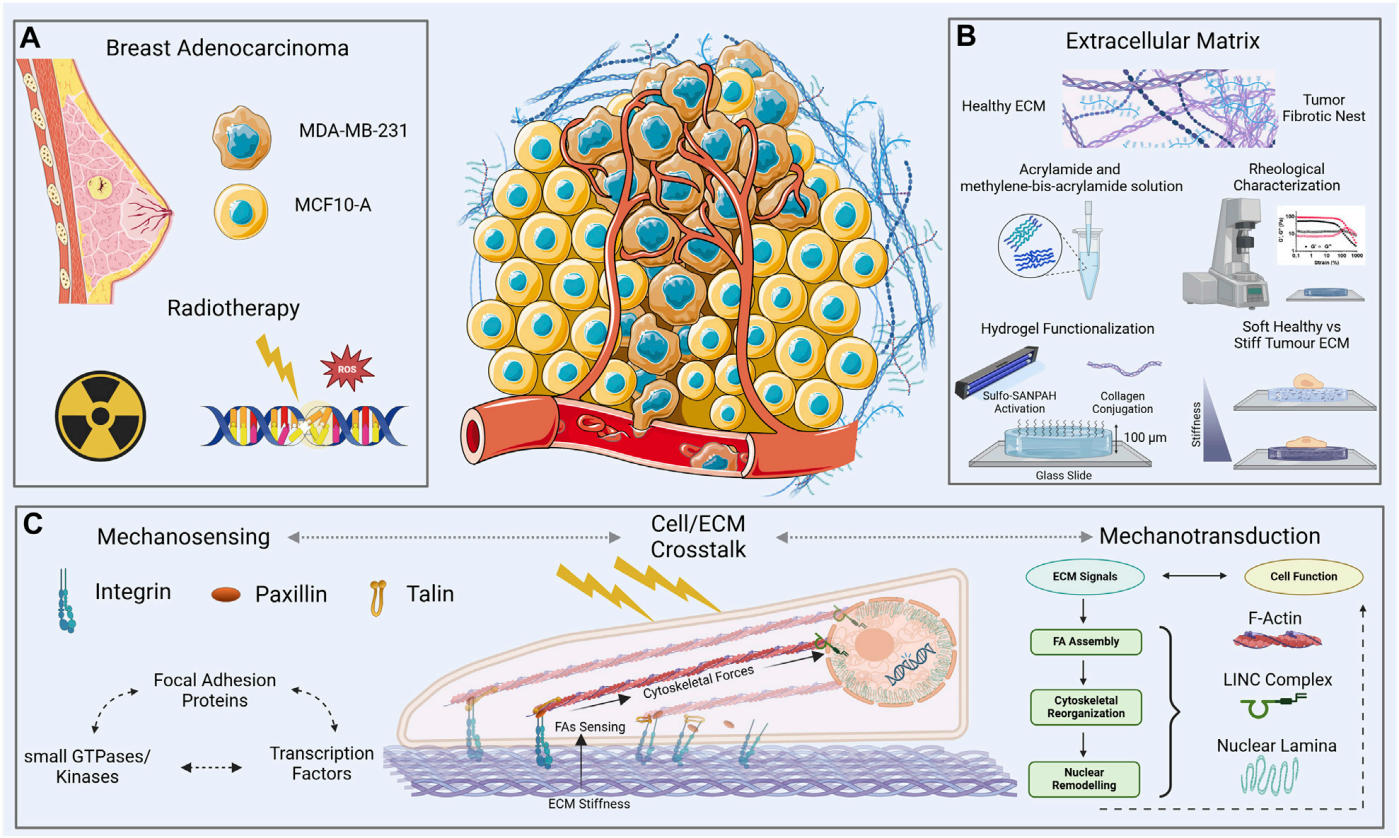
Overview of the experimental context—two cell lines, one healthy [normal mammary epithelial, MCF10A, and the other tumor (aggressive breast adenocarcinoma, triple negative, MDA-MB-231)] **(A)**, on two collagen functionalized-polyacrylamide (PAAm) substrates with a thickness of ∼100 μm **(B)**, and stiffnesses across a physio-pathological range of values [0.5–15 kPa, **(B)**]. The cell-ECM crosstalk **(C)** was evaluated through immunofluorescence and atomic force microscopy analysis 72 h after the exposure to two different doses of X-rays (2 and 10 Gy).

In this work we propose a mechanical characterization of both MCF10A, and MDA-MB-231 cell lines evaluated into physiological and pathological microenvironments after a conventional plan of clinical radiotherapy (Figures 1A,B). The aim of the work is to relate the mechanical behavior of both cell lines investigated to the molecular expression, availability and assembly of some key proteins involved in the mechanosensing and mechanotransduction processes upstream and downstream of RT treatment. Single cell immunofluorescence analysis was performed to elucidate the mechanotransduction processes initiated at the FAs and cytoskeletal levels (Figure 1C). This involved assessing the expression of paxillin and F-actin, as well as gauging the polymerization degree and anisotropy of cytoskeletal fibers. The morphological characterization was obtained through the development of a 3D-reconstruction algorithm, for the quantification of the global and nuclear volumes. Finally, atomic force microscopy (AFM) was used to measure single cells’ compressibility, in terms of Young’s modulus. The analysis was performed 72 h after irradiation with cells cultured on polyacrylamide (PAAm) substrates mimicking different stiffness for the physiological and tumoral microenvironment (Young’s modulus of 0.5 and 15 kPa). This experimental set-up allowed to investigate the interplay of both the dose-time factor and the key role operated by the mechanical environment in the mechanisms regulating the manifestation of cellular features (Figure 1B).

## 2 Materials and methods

### 2.1 Fabrication of substrates and mechanical characterization

Polyacrylamide substrates were prepared following a previously published method (Panzetta et al., 2017b; Frascogna et al., 2024), with some minor adjustments. Below a step-by-step description of the pursued methodology:

#### 2.1.1 Surface treatment of glass-bottom culture dishes

- The glass slides were coated with 20 mM NaOH solution. Upon evaporation, they were treated with 3-aminopropyltriethoxysilane (Sigma-Aldrich, St. Louis, MO, United States) for 20 min. The treated slides were then meticulously washed with water to remove excess reagent. Following this, they were covered with a 0.5% glutaraldehyde solution in PBS for 30 min. Finally, they were thoroughly washed with distilled water and air-dried. These glass slides were later affixed to WillCo dishes with a 22 mm aperture.

#### 2.1.2 Polyacrylamide mixture preparation

- A mixture of Polyacrylamide was created using 40% acrylamide and 2% methylene-bis-acrylamide in a phosphate-buffered saline (PBS) solution (A4058/M1533 Merck).
- Two different final concentrations were prepared: 2.5% acrylamide/0.15% methylene-bis-acrylamide and 8% acrylamide/0.1% methylene-bis-acrylamide, corresponding respectively to stiffness levels of 0.5 kPa and 15 kPa (Young’s modulus).

#### 2.1.3 Polymerization process

- Polymerization was initiated by adding 1/100th of the total volume of 10% ammonium persulfate and 1/1,000th of the total volume of N,N,N′,N′-tetramethylethylenediamide (TEMED, Sigma-Aldrich, St. Louis, MO, United States).
- The acrylamide/methylene-bis-acrylamide mixture was pipetted onto the treated glass-bottom culture dishes and covered with a 20-mm coverslip. The mixture volume was ∼30 μL in order to obtain hydrogels with a thickness of ∼100 μm.
- After 20 min, the coverslip was removed, and PBS was added to the dish.

#### 2.1.4 UV light exposure and sterilization

- The substrates were soaked with a penicillin–streptomycin solution overnight.
- PAAm’s substrates were exposed to UV light emitted by a germicidal lamp for 1 h to sterilize them.

#### 2.1.5 Collagen functionalization

- Substrates were functionalized with collagen using a bifunctional photoreactive crosslinker (sulfosuccinimidyl 6-(4′-azido-2′-nitrophenylamino) hexanoate, sulfo-SANPAH (Fischer Scientific, Loughborough, United Kingdom).
- The sulfo-SANPAH solution was diluted in double-distilled water at a final concentration of 0.2 mg/mL, placed on Polyacrylamide substrates, and exposed to 365 nm UV light for 10 min.
- After washing with PBS, the substrates were incubated with a solution of bovine type I collagen (Sigma-Aldrich, C4243) at a final concentration of 50 μg/mL in bi-distilled water for 1 h at 37°C.
- Finally, the samples were washed with PBS.

### 2.2 Cell lines and cell cultures

MCF10A cells (generously provided by Stefano Piccolo, AIRC Institute of Molecular Oncology, 20139 Milan, Italy) were cultured in Dulbecco’s Modified Eagle’s Medium/Nutrient Mixture F-12 Ham (Sigma, St. Louis, MO, United States). The medium was supplemented using 5% horse serum (HS), 1% L-glutamine (Sigma, St. Louis, MO, United States), 1% penicillin–streptomycin (Sigma, St. Louis, MO, United States), 0.1% insulin, 0.1% hydrocortisone and 0.1% epithelial growth factor (EGF).

MDA-MB-231 cells (generously provided by Francesco Paolo Cammarata, Institute of Molecular Bioimaging and Physiology, National Research Council IBFM-CNR, 90015 Cefalù, Italy) were cultured in the same base medium used for MCF10A. The medium was supplemented with 10% foetal bovine serum (FBS, Gibco, Eggenstein, Germany), 1% L-glutamine (Sigma, St. Louis, MO, United States) and 1% penicillin–streptomycin (Sigma, St. Louis, MO, United States).

### 2.3 External beam radiotherapy

Cell lines were exposed to X-rays delivered by external beam radiotherapy through the LINAC Synergy Agility system (ELEKTA), employing a 6 MV energy beam. Radiation doses of 2 and 10 Gy were delivered by 3D conformal radiation therapy (3D-CRT) treatment plans generated with the Monaco v5.11.03 treatment planning station by Elekta, as previously applied (Panzetta et al., 2017b; Frascogna et al., 2024). The selection of 2 Gy signifies the standard fractional dose commonly administered in oncological routine treatments, while 10 Gy aligns with the dosage typically employed as a post-operative boost treatment. In the implemented experimental arrangement, cells were placed in a WillCo dish between two solid water phantom slabs (ScandiDos Delta-4 Calibration Phantom), each with dimensions of 3 and 5 cm. Cells underwent irradiation from opposing fields through a 180° gantry rotation. 200 UM/min was chosen as the fixed dose rate. Doses were administered at the cell level on a uniform square field (20 × 20 cm^2^). After exposure, cells were cultured at 37°C and 5% CO_2_. Samples were fixed at room temperature after 72 h from radiotherapy using a 4% paraformaldehyde solution in 1× PBS for 20 min.

### 2.4 Immunostaining

MCF10A and MDA-MB-231 cells, fixed after 72 h from radiotherapy, were immunostained for the evaluation of cytoskeletal F-actin’s and Paxillin’s expression and for the analysis of nuclear and membrane volumes.

Cell membrane was stained for 30 min with Cell Tracker™ (C34565 Thermo Fisher Scientific) Red CMTPX (λ_excitation_ ∼ 577 nm, λ_emission_ ∼ 602 nm) at 1/1,000 dilution in PBS. Cell nuclei were stained in Hoechst 33342 (Sigma-Aldrich St. Louis, MO, United States) (λ_excitation_ ∼ 360 nm, λ_emission_ ∼ 460–470 nm) at 1/1,000 dilution in PBS.

Cytoskeletal F-actin and Paxillin were stained employing the following protocol: cells were permeabilized with 0.1% Triton X100 (Sigma-Aldrich, T9284, St. Louis, MO, United States in 1 ×  PBS for 15 min and blocked with 3% bovine serum albumin (BSA, A9418 Sigma-Aldrich St. Louis, MO, United States) at 0.1% dilution in Triton X100 in 1 ×  PBS for 1 h. Cells were stained for F-actin with Alexa Fluor 488 phalloidin (λ_excitation_ ∼ 488 nm, λ_emission_ ∼ 520 nm) at 1/200 dilution in 3% BSA—0.1% Triton X100 in 1 ×  PBS for 1.5 h.

Paxillin was stained using rabbit anti-paxillin monoclonal antibody for 1.5 h (Abcam, 1:250 dilution in PBS-BSA3%); then cells were incubated with Alexa546 goat anti-rabbit secondary antibodies (Life Technologies, A11035) for 1 h.

### 2.5 Image analysis

Individual cells’images were acquired using a Zeiss LMS-800 confocal microscope with a 40× immersion objective. Images were imported as z-stacks (file.TIFF) into Fiji-ImageJ software (NIH, Bethesda, MD, United States) to quantify, in each cell, the expression of cytoskeletal F-Actin and Paxillin. Signals of interest were projected onto the cellular adhesion plane. The definition of the region of interest (ROI) was obtained exploiting cell membrane signal to achieve cell spreading area, which was quantified using the “Measure” command in Fiji ImageJ (Supplementary Figure S1). Protein signals intensity was quantified as “Integrated Density” into the defined ROI. The quantification of Paxillin at the focal adhesions’ (FAs) level was obtained by only projecting the slices from the z-stack closer to the adhesion’s plane (corresponding to 3-4 slices), individuated on orthogonal views. The spreading area data was used to normalize Paxillin expression at the focal adhesions’ level (Paxillin density at FAs). Hence, the removal of the background was undertaken to create a faithful representation of the single cell’s structure using Imaris 3D (Oxford Instruments) software.

Imaris 3D allows the definition of a computational-3D model of the single cell, identified as “Surface.” This process involves pre-processing, segmentation, and labelling. Surface models allow the identification and quantification of a series of physical and morphological features within the cell (including area, volume, position, and elliptical features). The reconstruction is performed through an octal tree structure with a resolution level set to 0.3 μm. The adjustment of a manual threshold permits to modify the fluorescence signals’ intensity. Imaris 3D models were used to quantify the nuclear and membrane volumes (µm^3^) at the single cell level, for the different experimental conditions: this process allowed to normalized F-Actin total expression on the cell membrane volume. The normalization process guarantees the independence of the expression’s results from the cell specific morphological characteristics.

Furthermore, CSK’s fibers organization was investigated through the analysis of F-actin’s filaments median and total length. This data was obtained by a MatLab™ routine applied to the cytoskeletal projections on the adhesion’s plane (Rogge et al., 2017) (Supplementary Figures S2A, B). Moreover, filaments arrangement was examined on the same projections through a coefficient of anisotropy (in the range 0-1), extrapolated applying FibrilTool, an ImageJ plug-in to quantify fibrillar structures in raw microscopy images (Boudaoud et al., 2014) (Supplementary Figures S2C, D).

### 2.6 Statistical analysis

Results for each experimental condition were presented using boxplots, offering a visual depiction of individual sample distributions. The boxes, delineated by the first and third quartiles, encapsulate the median, with segments representing minimum and maximum values. The normality of data was assessed via the Shapiro–Wilk test (*p* < 0.05 indicating non-normal distribution). Statistical comparisons were conducted using Student’s unpaired *t*-test for normally distributed data, while non-normally distributed data underwent the nonparametric Kruskal–Wallis test. Statistical significance was assigned to differences with *p*-values < 0.05. In the graphical representations, statistical analyses are incorporated for two distinct categories of comparisons. The first type involves comparing control and irradiated samples within the same cell line, denoted by asterisks (*). The second analysis, indicated by hashtags (#), compares healthy and tumor cell lines under the same treatment conditions. The following graphical notation is adopted: *, #*p* < 0.05; **, ##*p* < 0.01; ***, ###*p* < 0.001. Additional details about the complete cross differences performed are provided in the Supplementary Material.

## 3 Results

### 3.1 Paxillin expression and FAs formation

**FIGURE 2:**
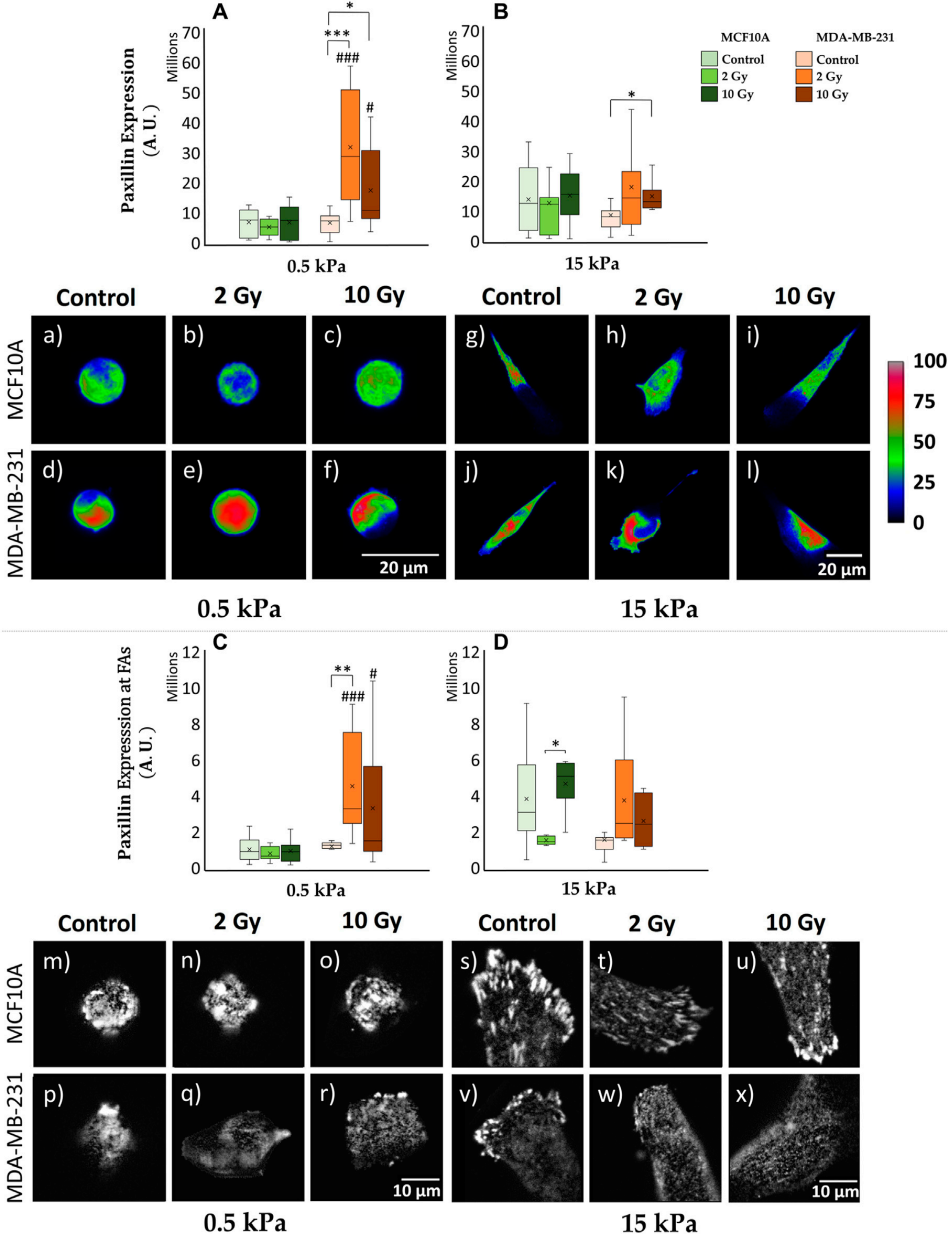
Differential mechanical response to radiation of non-tumorigenic (MCF10A; a–c, g–i, m–o, s–u) and malignant cells (MDA-MB-231; d–f, j–l, p–r, v–x) evaluated in terms of Paxillin expression **(A–D)** 72 h after RT. The analysis was performed for two stiffness of the ECM equal to 0.5 **(A, C)** and 15 kPa **(B, D)**. Cells were stained for paxillin, and single cells were acquired using a confocal microscope equipped with a ×40 objective. Paxillin expression was evaluated for each condition at the cytoplasmatic level [**(A, B)**; a-l] by projecting the entire single cell z-stack on the adhesion plane: the calibration bar fixes the look-up-table (“Rainbow RGB”) between 0 (black) and 100 (white) aiming to qualitatively describe paxillin expression (scale bar, 20 µm). Furthermore, paxillin expression was determined at the FAs level [**(C, D)**; m–x] by projecting only the slices (3-4 slices) from the z-stack specifically close to the adhesion plane: images (m–x) exhibit paxillin-rich-FAs in gray scale (scale bar, 10 µm). Significant statistical differences are reported: *, #*p* < 0.05; **, ##*p* < 0.01; ***, ###*p* < 0.001. *n* ≥ 10 for condition.

Cells establish a physical connection with the ECM through FAs, and the intricate components orchestrating this adhesion contribute to a dynamic interplay between intra and extracellular structures. Unraveling the intricate crosstalk between tumor cells and the ECM is imperative; understanding where this process is compromised provides insights into the altered mechanosensing mechanisms in cancer. The quantification of cell adhesion ability involves evaluating the spreading area, with detailed results provided in Supplementary Figure S1. As already reported (Frascogna et al., 2024), in control condition both cell lines increased their spreading area as substrate stiffness increased. When irradiated, MDA-MB-231 cells exhibit a greater spreading area in comparison with control cells. Then, to delve deeper into this process, we specifically targeted paxillin, a key protein of FA complex. As descripted in the materials and methods section, the expression of paxillin was evaluated both at the cytoplasmatic and at adhesion plane level: the former investigation aims to access at the cellular global availability of the protein in the diverse examined conditions; the latter allows to quantify the effective recruitment of paxillin at the FAs complex, and its actual involvement into the process of cell adhesion to the substrate. The cytoplasmic expression of paxillin in MCF10A cells exhibits no significant variation between 0.5 kPa and 15 kPa for non-irradiated cells (control conditions) (Figures 2A,B,a,g; Supplementary Figures S3A, B) and across different absorbed doses of ionizing radiation on a fixed substrate stiffness (Figures 2A,B,b,c,h,i; Supplementary Figures S3A, B). However, at the cell-substrate interface, specifically within assembled FAs, a significant (*p* < 0.05) reduction in paxillin expression is noted for control MCF10A cells cultured on 0.5 kPa compared to those on 15 kPa (Figures 2C,D,m,s; Supplementary Figures S3C, D). This behavior strongly suggests the discerning ability of MCF10A cells to perceive and respond to substrates of different stiffness, showcasing their active mechano-receptive machinery. Notably, differences in paxillin expression at the FAs level for varying radiation doses are not statistically significant for MCF10A on both substrates (Figures 2C,D,n,o,t,u; Supplementary Figures S3C, D). In contrast, MDA-MB-231 cells exhibit a distinctive behavior, indicative of alterations in mechanosensing-related structures: adenocarcinoma cells display no significant differences on controls in terms of paxillin absolute expression between 0.5 and 15 kPa (Figures 2A,B,d,j; Supplementary Figures S3A, B). The absolute cytoplasmic expression of paxillin shows a significant increase on 0.5 kPa (Figures 2A,d–f; Supplementary Figure S3A) for both 2 Gy (*p* < 0.001) and 10 Gy (*p* < 0.05) irradiated samples, with a similar trend observed on 15 kPa (Figures 2B,j–l; Supplementary Figure S3B), where statistical differences are observed only between control and 10 Gy (*p* < 0.05). This data should be referred to a distinct dose-dependent increase of cellular volume on every ECM (*p* < 0.001) for both 2–10 Gy, as even highlighted by the box-plots flattening for paxillin concentration (expression normalized on cell volume) (Supplementary Figures S3A, B). Contrary to the healthy cell line, breast adenocarcinoma cells MDA-MB-231 show no significant increase of paxillin local expression at FAs between 0.5 and 15 kPa for controls (Figures 2C,D,p,v; Supplementary Figures S3C, D). The softer ECM reveal the existence of a peculiar increase in paxillin local production moving from control to 2 Gy (*p* < 0.01), with no evidence of dissimilarity for the higher dose of 10 Gy or for increasing stiffness of the substrate at 15 kPa (Figures 2C,D,p–r,v–x; Supplementary Figures S3C, D).

### 3.2 F-actin expression and CSK organization

**FIGURE 3:**
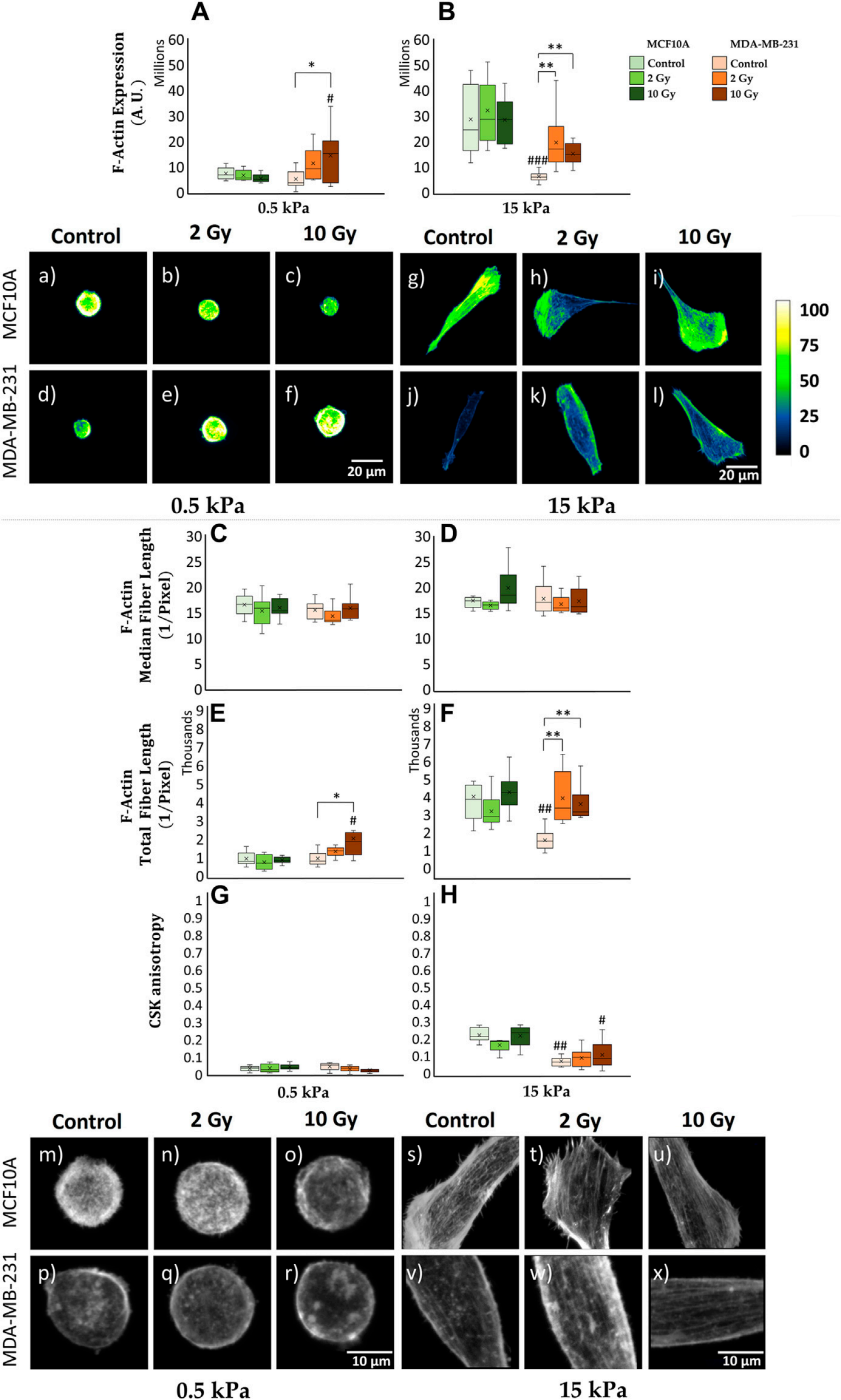
Differential mechanical response to radiation of non-tumorigenic (MCF10A; a–c, g–i, m–o, s–u) and malignant cells (MDA-MB-231; d–f, j–l, p–r, v–x) evaluated in terms of cytoskeletal F-actin expression **(A, B)**, median **(C, D)** and total **(E, F)** fiber length and anisotropy **(G, H)** 72 h after RT. The analysis was performed for two stiffnesses of the ECM equal to 0.5 **(A, C, E, G)** and 15 kPa **(B, D, F, H)**. Cells were stained for F-actin with Alexa Fluor 488 phalloidin, and single cells were acquired using a confocal microscope equipped with a ×40 objective. F-actin expression was evaluated for each condition at the cytoplasmatic level [**(A, B)**; a-l] by projecting the entire single cell z-stack on the adhesion plane: the calibration bar fixes the look-up-table (“Green fire Blue”) between 0 (black) and 100 (white) aiming to qualitatively describe F-actin expression (scale bar, 20 µm). The building of cytoskeletal fibers [**(C–H)**; m–x, scale bar, 10 µm] was characterized by the analysis of F-actin median/total polymerization **(C–F)** (Rogge et al., 2017) and organization through the definition of a coefficient of anisotropy **(G, H)** defined in the range between 0 (completely disordered fibers) and 1 (parallel fibers) (Boudaoud et al., 2014). Significant statistical differences are reported: *, #*p* < 0.05; **, ##*p* < 0.01; ***, ###*p* < 0.001. *n* ≥ 10 for condition.

The transduction of mechanical stimuli into biochemical responses passes through the mediation role operated by the CSK. Cellular mechanosensing is tuned by actin-generated cytoskeletal tension that balances external with internal mechanical forces. FAs are firmly anchored to actin stress fibers, resulting into a bidirectional pathway which regulates cell dynamical behavior as a response to ECM stiffness. Hence, the investigation of CSK’s rearrangements passes through the analysis of F-actin expression, indicating the amount of actin protein involved into the assembly of actin filaments (Figures 3A,B,a–l). F-actin expression shows for MCF10A the existence of a significant (*p* < 0.001) increase on 15 kPa when compared to 0.5 kPa for control conditions (Figures 3A,B,a,g). This finding aligns with the observations related to cell’s spreading (Supplementary Figure S1) and FAs formation (Figure 2). No statistically significant difference emerges from the analysis of healthy irradiated cells, for fixed substrate’s stiffness (Figures 3A,B,b,c,h,i). The same results are observed for MCF10A in the case of F-actin concentration, after normalizing cytoskeletal integrated density on cellular volume (Supplementary Figure S3E, F). MDA-MB-231 shows no significant difference in terms of F-actin expression for not irradiated samples cultured on distinct substrate’s stiffness (Figures 3A,B,d,j), consistently with the previous discussed analysis on paxillin-FAs (Figure 2). On the contrary, F-actin fluorescence significantly increases for 10 Gy irradiated cells (*p* < 0.05) on 0.5 kPa (Figures 3A,e,f); similarly, on 15 kPa (Figures 3B,k,l), cancer cells exhibit a marked escalation for both 2 Gy (*p* < 0.01) and 10 Gy (*p* < 0.01), indicating an elevated radio-induced tension at actin stress fibers. Furthermore, utilizing the F-segment MatLab™ routine (Rogge et al., 2017), we gained insights into the cytoskeletal structure and organization (Figures 3C–H,m–x). Not irradiated MCF10A cells on 15 kPa exhibited significantly higher cytoskeletal polymerization (F-actin total fiber length) than on 0.5 kPa (*p* < 0.001) (Figures 3E,F,m,s). There were no discernible differences in polymerization observed in irradiated samples on both physiological and cancerous ECMs (Figures 3C–F,n,o,t,u). In contrast, MDA-MB-231, while showing no statistical distinctions in controls (Figures 3C–F,p,v), attributable to a compromised mechanosensing apparatus, demonstrated a significant cytoskeletal polymerization when irradiated at 10 Gy (*p* < 0.05) on 0.5 kPa (Figures 3E,r) and for both 2 Gy (*p* < 0.01) and 10 Gy (*p* < 0.01) on 15 kPa substrate (Figures 3F,w,x). Moreover, CSK fibers isotropy was extrapolated applying FibrilTool, an ImageJ plug-in to quantify fibrillar structures in raw microscopy images (Boudaoud et al., 2014). Both normal and cancer cells highlighted the lack of any CSK isotropy when seeded on 0.5 kPa, for every dose of IR (Figures 3G,m–r). Conversely, on 15 kPa, MCF10A displays a significant increase in terms of cytoskeletal organization (*p* < 0.001), with higher isotropy of CSK fibers than what observed on the softer ECM (Figures 3H,s–u). In this context, RT seems not to affect F-actin arrangements on healthy irradiated specimens. On the same stiffer substrate, MDA-MB-231 shows, for both controls and irradiated cells, lower CSK ordering with respect to their healthy counterpart (Figures 3H,v–x), suggesting the existence of a potential greater metastatic capacity for those cells who survived RT after 72 h.

### 3.3 AFM and cell mechanics

**FIGURE 4:**
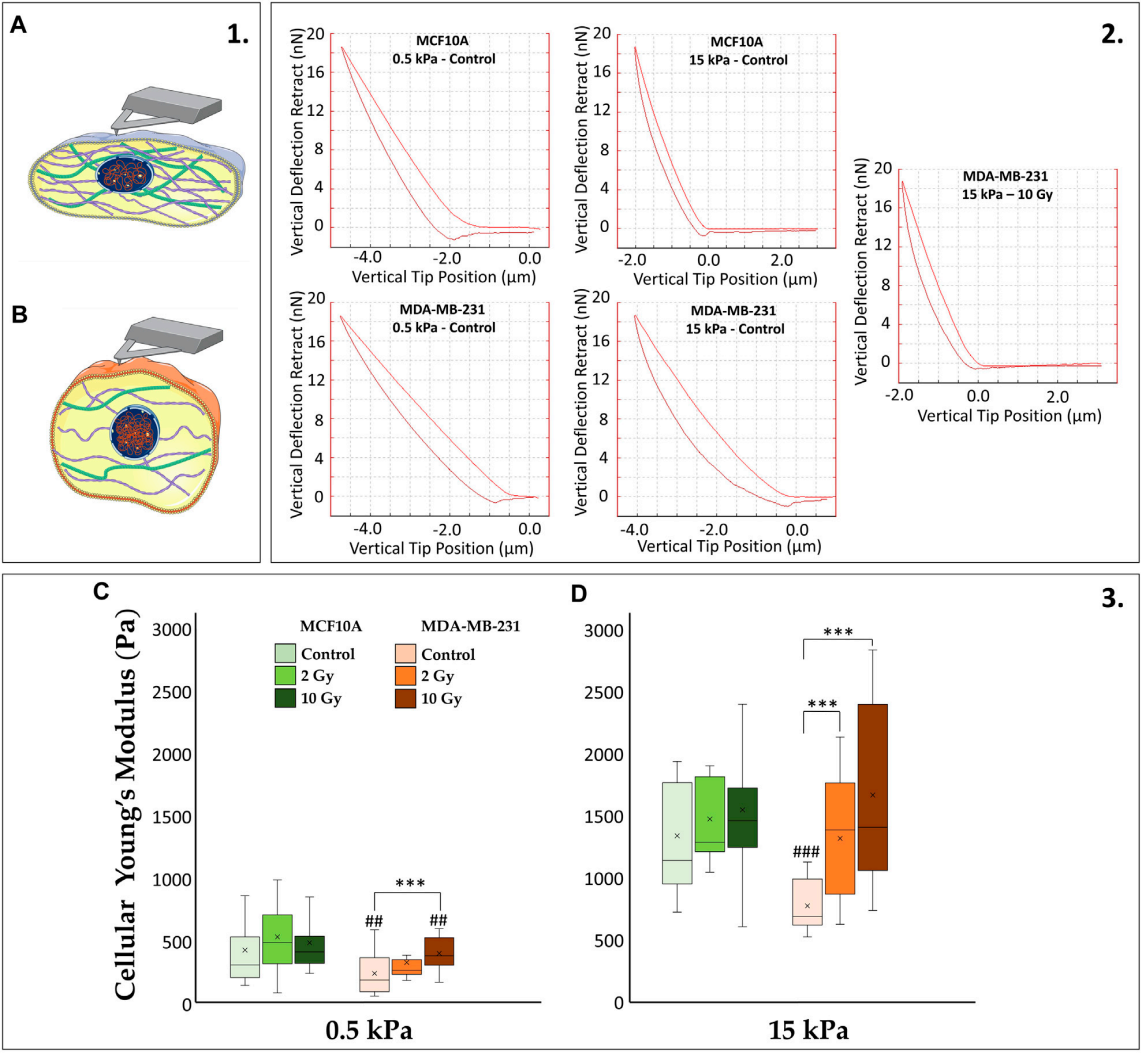
(1) The cartoon emphasizes how stiffer cells **(A)** differ from softer ones **(B)** in terms of more structured CSK whose tension consequently affects cytoplasmatic and nuclear morphologies. (2) The diverse cellular mechanics are reported, for representative conditions, in terms of AFM deflection curves. The AFM (operating in Tapping mode) quantifies the stiffness of the biological sample by recording the vertical force between the tip and the cell membrane as a function of the vertical tip position: the more the “pushing in” phase approaches the “pulling out” phase of the curve, the stiffer the cell will be (increasing cellular Young’s Modulus). (3) Cellular Young’s Modulus, measured through atomic force microscopy (AFM) for the two cell lines, MCF10A and MDA-MB-231, fixed at 72 h after RT, evaluated for two different stiffness of the ECM at 0.5 kPa **(C)** and 15 kPa **(D)**. Significant statistical differences are reported: *, #*p* < 0.05; **, ##*p* < 0.01; ***, ###*p* < 0.001. *n* ≥ 10 for condition.

In our investigation, we have observed distinct characteristics in the ability of healthy and tumor cell lines to sense mechanical signals from the microenvironment under control conditions. Furthermore, the differential impact of radiation on paxillin expression and cytoskeletal fiber arrangement in these two cell lines has been evident. It is reasonable to anticipate that such rearrangements correspond to diverse mechanical properties in the cells. To explore this, we employed AFM to quantitatively assess the Young’s modulus of the cells (Figure 4). MCF10A and MDA-MB-231 demonstrate a substantial increase (∼72%) in cellular stiffness (i.e., Young’s modulus) when transitioning from 0.5 to 15 kPa substrates (*p* < 0.001). Despite these similarities, the absolute values differ considerably, with MDA-MB-231 being more deformable. This is in line with their capacity to deform for invading adjacent tissues compared to their normal epithelial counterparts. Differences in the mechanoreceptive machinery between the two cell lines become evident in irradiated samples. MCF10A shows no significant differences on both 0.5 and 15 kPa between irradiated and control samples. Conversely, MDA-MB-231 exhibit a dose-dependent increase in stiffness, produced by the previously mentioned rise in paxillin and F-actin expression. On 0.5 kPa, MDA-MB-231 shows a significant increase of the Young’s modulus only at 10 Gy (*p* < 0.001), while on the pathological ECM, cancer cells display significant stiffening for both 2 and 10 Gy (*p* < 0.001) absorbed doses compared to controls.

### 3.4 Cellular and nuclear volumes

**FIGURE 5:**
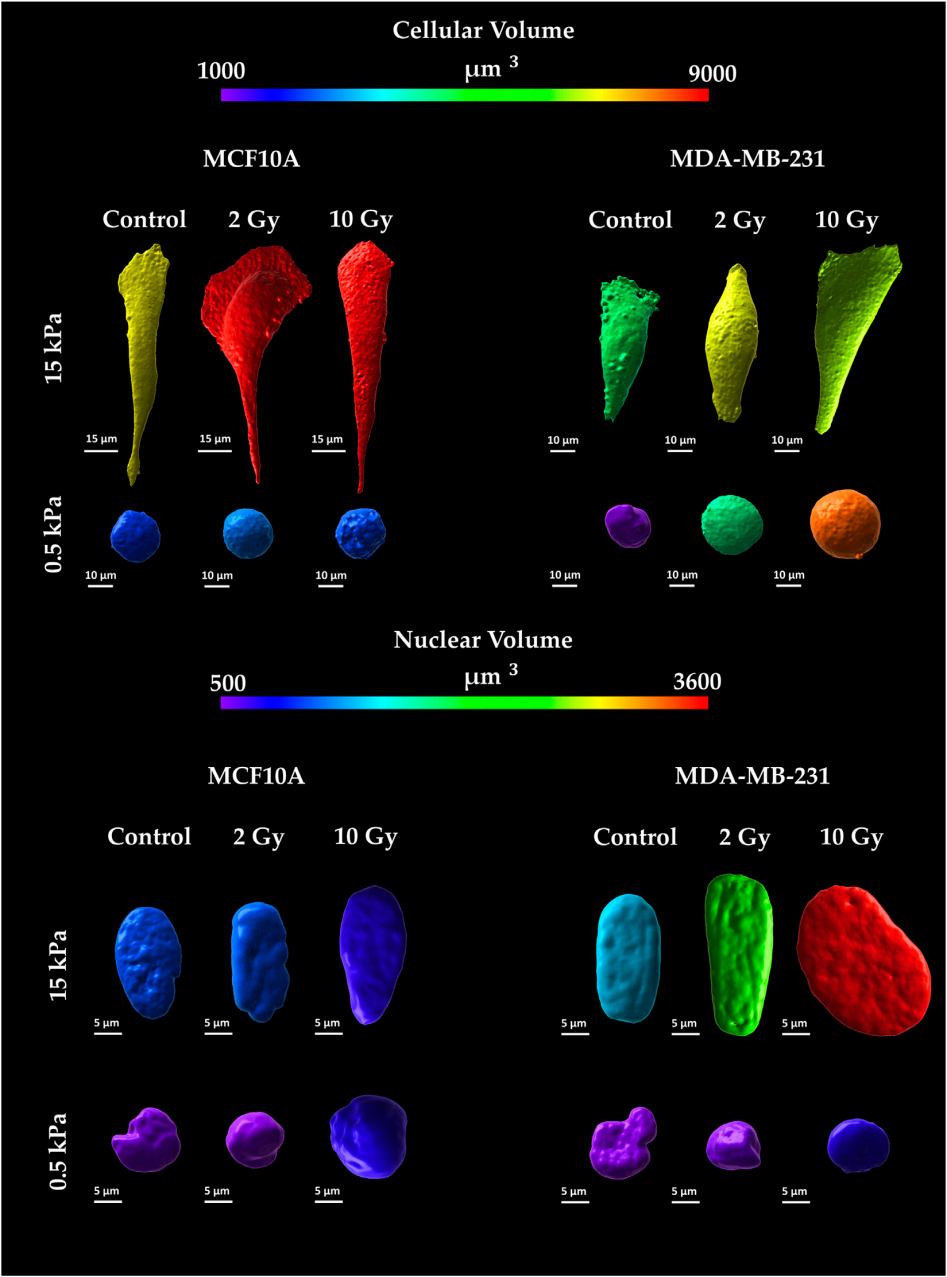
Representative images of cellular and nuclear volumes for the two cell lines, MCF10A and MDA-MB-231, fixed at 72 h after RT, evaluated for two stiffness of the ECM equal to 0.5 and 15 kPa. Cellular membranes were stained for Cell Tracker™ (C34565 Thermo Fisher Scientific) Red CMTPX; Cell nuclei were stained in Hoechst 33342 (Sigma-Aldrich St. Louis, MO, United States). Single cells were acquired using a confocal microscope equipped with a ×40 objective. Volumes were reconstructed using a computational algorithm implemented in Imaris 3D software (Oxford Instruments) through the definition of “surfaces” structures as described in the Image Analysis section. The calibration bar fixes the look-up-table (“Spectrum”) between 1,000 µm^3^ (violet) and 9,000 µm^3^ (red) aiming to qualitatively describe cytoplasmatic volumes. Likewise, nuclear volumes are described using the same look-up-table fixed in the range between 500 µm^3^ (violet) and 3,600 µm^3^ (red).

**FIGURE 6:**
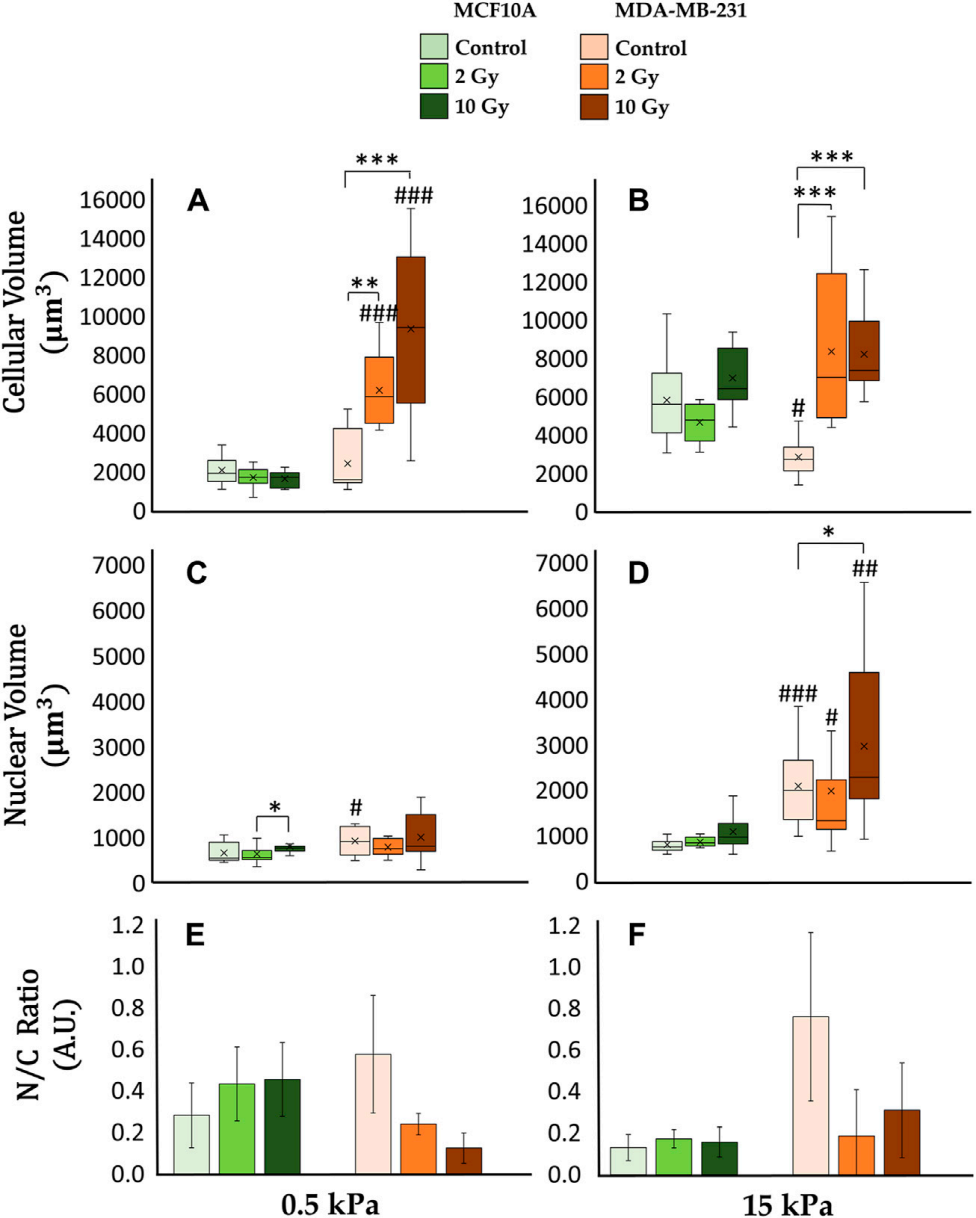
Differential mechanical response to radiation of non-tumorigenic (MCF10A; a–c, g–i, m–o, s–u) and malignant cells (MDA-MB-231; d–f, j–l, p–r, v–x) evaluated in terms of modifications of the total **(A, B)** and nuclear **(C, D)** volume 72 h after RT. The analysis was performed on samples from Figure 5 for two different stiffness of the ECM at 0.5 kPa **(A, C, E)** and 15 kPa **(B, D, F)**. Significant statistical differences are reported: *, #*p* < 0.05; **, ##*p* < 0.01; ***, ###*p* < 0.001. *n* ≥ 10 for condition. Clustered Columns **(E, F)** report the ratio between the nuclear and the total cytoplasmatic volume as N/C ratio. Error bars are obtained from the propagation of statistical errors of nuclear volume $\left(X\pm \sigma_{X}\right)$ and cellular (total) volume $\left(Y\pm \sigma_{Y}\right)$ , given by the formula $\sigma \left(\frac{X}{Y}\right)=\frac{1}{Y^{2}}\sqrt{Y^{2}\sigma_{X}^{2}+X^{2}\sigma_{Y}^{2}}$ .

The confocal microscopy acquisitions allowed the optimizations of a computational algorithm for the reconstruction of 3D models at the single cell level. This process ensured the quantification of both cellular and nuclear volumes (Figures 5, 6), to further discuss the entwined role of ECM mechanics and RT on altering cellular morphologies. In this context, not irradiated MCF10A display a significant increase of cytoplasmatic volume (Figures 6A,B), passing from 0.5 to 15 kPa substrates (*p* < 0.005). This result finds accordance with the largely discussed active mechanosensing operated by normal epithelial cells: the existence of a linear growth of cellular volume in control conditions, joins with the augmentation of spreading-area (Supplementary Figure S1) as well as with both the rising cytoskeletal recruitment and the paxillin-focal adhesion buildup (Figure 2). Both substrates highlighted, for normal epithelial cells, the absence of significant differences from the control onto irradiated samples in terms of alterations of the cellular cytoplasmatic volume (Figures 6A,B). The analysis on 3D-reconstructed nuclear volumes (Figures 6C,D) displays other additional intriguing information: despite cytoplasm, nuclear volumes do not show any morphological variation when passing from 0.5 to 15 kPa in the case of MCF10A cells. In line with the correlation between the tensile state of cytoskeletal fibers and nuclear morphology in normal epithelial cells, a significant increase in nuclear volume is observed only in the pathological microenvironment for 10 Gy irradiated samples (*p* < 0.05). Conversely, no type of remarkable alteration is seen on the physiological ECM for irradiated samples with respect to the control. On the other hand, adenocarcinoma cells volumes (Figures 6A,B) exhibit no significant differences on controls between 0.5 and 15 kPa. Unlike their normal epithelial counterpart, MDA-MB-231 cells displays the existence of a linear dose-dependent increase of cellular cytoplasmatic volume, on every investigated microenvironment (*p* < 0.001 for both 2 and 10 Gy). Furthermore, not irradiated adenocarcinoma cells increase their nuclear volume when passing from 0.5 to 15 kPa (*p* < 0.01): this behaviour points out how neoplastic cells turn out to be more susceptible of cytoskeletal tension over nucleus, despite their normal counterparts (MCF10A), when passing to a stiffer ECM; at the same time, RT seems not to significantly affect additional increments of cancer cells nuclear volume with respect to the control, on every substrate.

## 4 Discussion

The intricate interplay between cellular homeostasis and pathological conditions, such as cancer, lifts upward the communication between cells and their ECM (Nazemi and Rainero, 2020). While extensive research has historically focused on biochemical pathways, recent attention has shifted towards exploring the complex realm of mechanical signalling pathways. Cellular function hinges on microenvironmental cues, with cancer inducing not only alterations in cellular phenotype but also a broader transformation that encompasses changes in the ECM (Najafi et al., 2019; Panciera et al., 2020). The progression of cancer involves a series of interconnected events, including enhanced cell movement, alterations in adhesion receptors and cytoskeletal architecture, deregulation of cytoskeletal dynamics mediators, and changes in migratory properties (Lark et al., 2005; Desgrosellier and Cheresh, 2010). These changes in cellular behavior collectively contribute to the aggressive phenotype of neoplastic cells and their ability to invade surrounding tissues and metastasize to distant sites. Our investigation delves into the intricate processes of mechanosignalling and mechanotransduction, mapping the trajectory of signals from external to internal cellular domains. Starting at FAs, mechanical cues traverse along actin stress fibers, influencing cellular stiffness (Provenzano and Keely, 2011; Goldmann, 2012). In this dynamic interplay, tension exerted by the CSK results in compressive forces that alter nuclear morphology impacting the spatial organization of the genetic material (Edens et al., 2013; Schreiber and Kennedy, 2013; Tamura et al., 2013). The comprehensive exploration of both CSK and cellular morphology provides valuable insights into a more profound comprehension of cancer progression, guiding the orientation and assessment of clinical outcomes. In this study, we specifically assessed the interplay between ECM properties and RT on cell mechanical identity. Commencing our examination with an evaluation of paxillin expression, a crucial adaptor protein involved in FA assembly and maturation, residing both in the cytoplasm and at the basal membrane. The distinct localizations of paxillin bear significance: when associated with the membrane, paxillin correlates with cell adhesion, while its presence in the cytoplasm or nucleus is involved in creating extra FAs or participating in transcription pathways, mediating both extranuclear and intranuclear signaling (Sen et al., 2012). Healthy epithelial cells (MCF10A) exhibit the ability to accurately perceive substrate stiffness in line with existing literature (Goldmann, 2012; Jansen et al., 2015; Panzetta et al., 2017c; La Verde et al., 2021; Frascogna et al., 2024). This is supported by the observed increase in paxillin expression at FA level when transitioning from a soft to a stiff material under control conditions (i.e., not irradiated). Interestingly, irradiation appears not to impact cytoplasmic paxillin availability, maintaining steady levels for fixed ECM mechanical properties. However, distinct radiation doses induce local fluctuations in paxillin expression at membrane-substrate interaction sites (Figures 2C,D). Conversely, adenocarcinoma cells (MDA-MB-231) exhibit a distinctive response: they respond to stiffening transitions displaying unvaried paxillin levels at FAs sites, indicative of altered mechanosensing-related structures (Panzetta et al., 2019; Frascogna et al., 2024). The heightened motility of cancer cell, reliant on the rapid assembly and disassembly of FAs, and intricately intertwines with a dynamic cellular metabolism, which tunes energy source generation for driving cell migration through heterogeneous ECM (Kondo et al., 2021; Zanotelli et al., 2021). Recently, a previously undisclosed role has been also unveiled, indicating that the nuclear localization of paxillin acts as a transcription factor for SRC, leading to angiogenesis and tumor growth (Dong et al., 2009; Noh et al., 2021). The analysis of post irradiated samples, exhibited for cancer cells the existence of a significant dose-dependent increase in cytoplasmic paxillin expression, reflecting elevated paxillin levels at FAs sites (Figures 2C,D). These results align with the increased adhesion of MDA-MB-231 cells on both substrates described by the cell spreading investigation and by other previous researches (Nguemgo Kouam et al., 2019; Frascogna et al., 2024). These findings open to important outcomes in terms of understanding RT impact on radiation-resistant tumor metastasis especially if coupled with the altered cellular metabolism following IR that can further augment the energy supply for cancer cells (Yang et al., 2023). To understand the mechanical pathways related to this potential increased adhesion on adenocarcinoma cells after RT, we then turn our focus to the CSK. Tumor progression involves cells gaining increased mobility, invading nearby tissues, and migrating to distant sites. The aggressive behavior of cancer cells is linked to modifications in CSK architecture and in the dynamics of its mediators (Wu et al., 2020). These changes collectively result in alterations in the mechanical features of cancer cells: heightened aggression and invasiveness correspond to reduced cell stiffness. Moreover, recent research indicates that cell migration is influenced by nuclear stiffness. A study on the mechanics of cancer cell invasion through 3D extracellular matrices has shown that nuclear softening of MCF7 cells leads to an increase in invasion depth (Fischer et al., 2020). In this context, stiffness not only serves as an indicator of invasive potential but also offers insights into the effectiveness of the RT treatments. Remarkably, the healthy cell line exhibited lower sensitivity to IR, and the observed biophysical effects on the actin network and cellular stiffness were negligible. Contrary, for cancer cells, the analysis of F-actin fibers reveals the existence of an elevated radio-induced polymerization and tension at the stress fibers level; this result is further strengthened by AFM measurements displaying, especially on the pathological microenvironment, a notable cancer cells stiffening, linear with the absorbed RT dose (Figures 4C,D). This last observation aligns with previous published data regarding the motility of MDA-MB-231 (Panzetta et al., 2020): cancer cells who survived treatment, after 72 h from irradiation, exhibited a dose-dependent decrease in migration velocity. These findings open the way to a possible mitigation of radiation-resistant adenocarcinoma cells aggressivity after RT. Our investigation went deeper into the exploration of cellular altered morphologies. As stress fibers transmit tension forces within the cell, this mechanical signaling influences cell deformability, with impact on both cellular and nuclear morphology. The dynamic interplay between stress fiber tension and nuclear configuration reveals a critical link in understanding how mechanical forces are intricately interconnected, shaping the overall cellular response to external stimuli. The correct mechanosensing operated by normal epithelial cells prompts an expansion of the cell cytoplasmic volume in response to a stiffer ECM (Figures 6A,B). This is evident in the linear growth of cellular volume under control conditions that aligns coherently with the found increased spreading area, heightened cytoskeletal recruitment, and the formation of FAs (Mullen et al., 2014; Chen et al., 2015). In contrast, irradiated samples of normal epithelial cells exhibit a resilience to ionizing radiation, displaying no significant alterations in cytoplasmic volume compared to controls, regardless of substrate stiffness (Figures 6A,B). This resilience suggests a robust response to irradiation in terms of cellular mechanics. Shifting focus to the analysis of the breast adenocarcinoma cell line, it is evident that even under control conditions, the regulation of cell volume does not align with the proper mechano-perception of the changing ECM properties: cancer cells, in fact, do not show any significant increase of cytoplasmatic volume passing from a soft to a stiff substrate. Moreover, contrary to the healthy cell line, tumor cells exhibit heightened sensitivity to ionizing radiation. The increased cytoskeletal tension and actin polymerization triggered by irradiation contribute to a notable dose-dependent increase in cellular volume. In this context, reconstructing nuclear morphologies was essential for a deeper understanding of cell response to RT. Despite the stiffened cytoskeletal structure on the stiff substrate compared to the soft, the non-irradiated healthy cell line maintains a consistent nuclear volume, crucial for proper cell function and disease prevention on every ECM (Figures 6C,D). In contrast, the cancerous line displays larger nuclei on both substrates, with an increasing trend from soft to stiff matrix. Changes in nuclear shape can result from modifications in cell-generated mechanical forces or alterations in nuclear structural components, such as the nuclear lamina and chromatin, which act to counterbalance these forces (Singh and Lele, 2022). Radiation therapy induces a dose-dependent increase in nuclear volume for both cell lines. Nevertheless, the equilibrium between nuclear volume growth and cytoplasmic volume growth differs between the two cell lines. The nuclear-to-cytoplasmic (N/C) ratio, a critical determinant for proper cellular functioning, must adhere to specific physiological requirements (Balachandra et al., 2022). This ratio serves as a valuable metric for assessing malignancy in histology. Under control conditions, on both substrates, the N/C ratio is higher for the tumor line compared to the healthy line. RT affects this ratio, but in opposite directions for the two cell lines. Specifically, the N/C ratio experiences an increase for MCF10A and a decrease for MDA-MB-231 under the action of IR (Figures 6E,F). The effects on cellular and nuclear morphology need further investigation to better understand RT impact on the chromatin condensation state and, consequently, on the activation of specific transcriptional pathways in relation to the ECM mechanics.

## 5 Conclusion

In this study, a significant investigation was presented regarding the impact of RT on biophysical properties of breast adenocarcinoma cells. Our findings revealed that the effects of IR on cell adhesion and mechanics are contingent upon both the dose administered and the stiffness of the substrate on which the cells are cultured. Specifically, we observed dose-dependent alterations in cellular adhesion and mechanics, with varying substrate stiffness leading to distinct responses. This underlines the intricate relationship with microenvironmental mechanical cues that are essential for triggering tumorigenesis in normal cells and that constitutes a fundamental pursuit in the field of cancer biology.

One notable outcome of our investigation was the observed shifts in paxillin expression following exposure to IR. In particular, the increase in paxillin expression on both substrate for the tumor line may be beneficial as it corresponds to more structured FAs, thereby resulting in reduced motility rates. This modulation correlated with changes in cytoskeletal Young’s modulus, indicating a direct mechanistic link between radiation exposure and cellular biomechanics.

Furthermore, our study highlighted the consequential impact of these radiation-induced alterations on nuclear morphology. These variations are indicative of underlying shifts in cellular homeostasis and functional adaptations in response to radiation-induced stress.

Overall, our findings underscore the intricate interplay between IR, ECM, and cellular mechanical identity in conventional radiotherapy treatments of breast adenocarcinoma. This deeper understanding of the mechanobiological responses to radiation exposure may have implications for optimizing therapeutic strategies, particularly in the context of radiotherapy and radio-chemotherapy treatments.

## Data Availability

The raw data supporting the conclusion of this article will be made available by the authors, without undue reservation.
