# Supplementary material for: Impact of ionizing radiation on cell-ECM mechanical crosstalk in breast cancer
Source: Front Bioeng Biotechnol. 2024 Jun 6;12:1408789. doi: 10.3389/fbioe.2024.1408789 (PMC11187264; doi:10.3389/fbioe.2024.1408789)
Supplement: Supplementary file 1 [file DataSheet1.docx]

Supplementary Material

Impact of Ionizing Radiation on Cell-ECM Mechanical Crosstalk in Breast Cancer

Rocco Mottareale^1,2,†^, Crescenzo Frascogna^3,4,†^, Giuseppe La Verde^1^, Cecilia Arrichiello^5^, Paolo Muto^5^, Paolo A. Netti^3,4,6^, Sabato Fusco^3,7,*^, Valeria Panzetta^3,4,6,§^, Mariagabriella Pugliese^1,§^

^1^Department of Physics “E. Pancini”, University of Naples Federico II, Via Cinthia, 80126 Naples, Italy.

^2^CNR-ISASI, Institute of Applied Sciences and Intelligent Systems E. Caianiello, Pozzuoli, Italy.

^3^Center for Advanced Biomaterials for Healthcare @CRIB, Italian Institute of Technology, Largo Barsanti e Matteucci 53, 80125 Naples, Italy.

^4^Department of Chemical, Materials and Production Engineering, University of Naples Federico II, Piazzale Vincenzo Tecchio, 80125 Naples, Italy.

^5^Radiotherapy Unit, Istituto Nazionale Tumori-IRCCS-Fondazione “G. Pascale”, Via Semmola, 53, 80131 Naples, Italy.

^6^Interdisciplinary Research Centre on Biomaterials CRIB, University of Naples Federico II, Piazzale Vincenzo Tecchio, 80125 Naples, Italy.

^7^Department of Medicine and Health Sciences “V. Tiberio”, University of Molise, Via Cesare Gazzani, 86100 Campobasso, Italy.

^†^These authors contributed equally to this work and share first authorship

^§^These authors contributed equally to this work and share last authorship

*** Correspondence:** Sabato Fusco (e-mail:sabato.fusco@unimol.it)

## Supplementary Figures

**
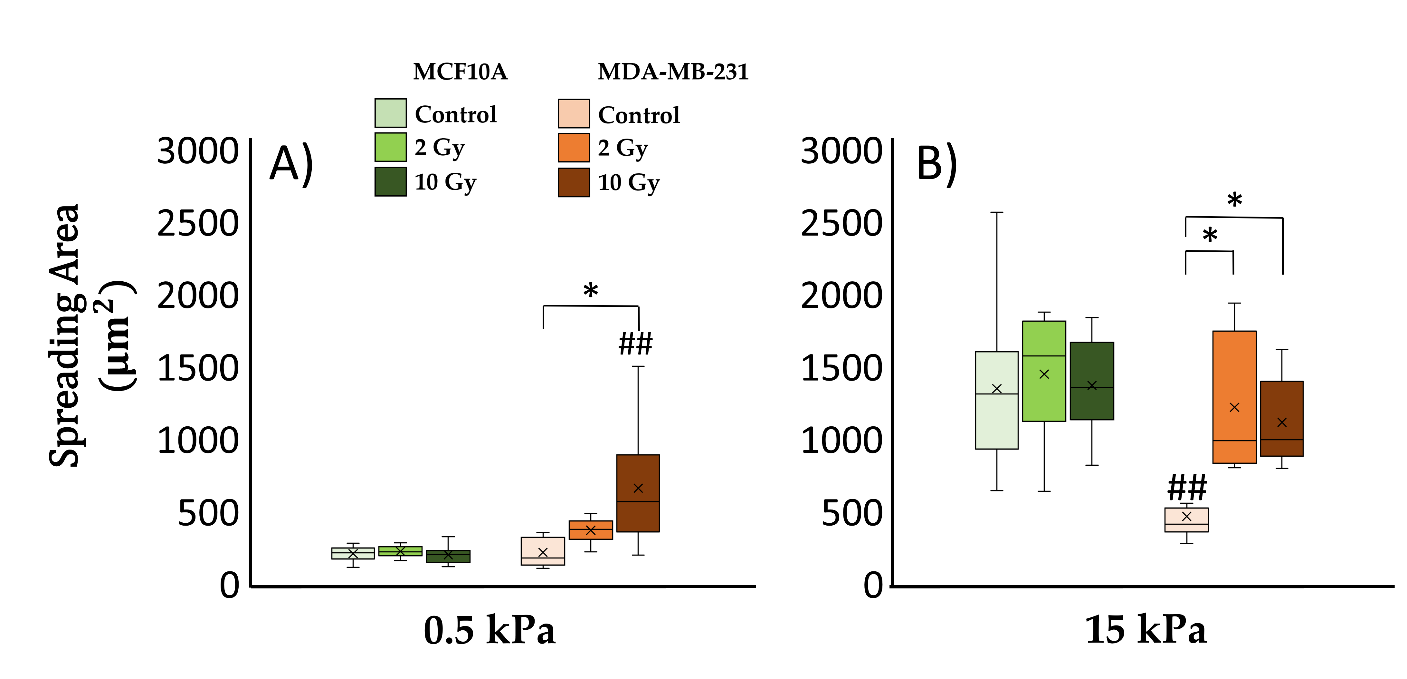
**

**Supplementary Figure 1.** Box plots (mean, median, interquartile range, and outliers) of cellular spreading area  for the two cell lines, MCF10A and MDA-MB-231, fixed at 72 h after RT, evaluated for two different stiffness of the ECM at 0.5 kPa (A) and 15 kPa (B). Cells’ membranes were stained with Cell Tracker™ (C34565 Thermo Fisher Scientific) Red CMTPX. Acquisitions were performed using a confocal microscope equipped with a ×40 objective. n ≥ 10 for condition.

**
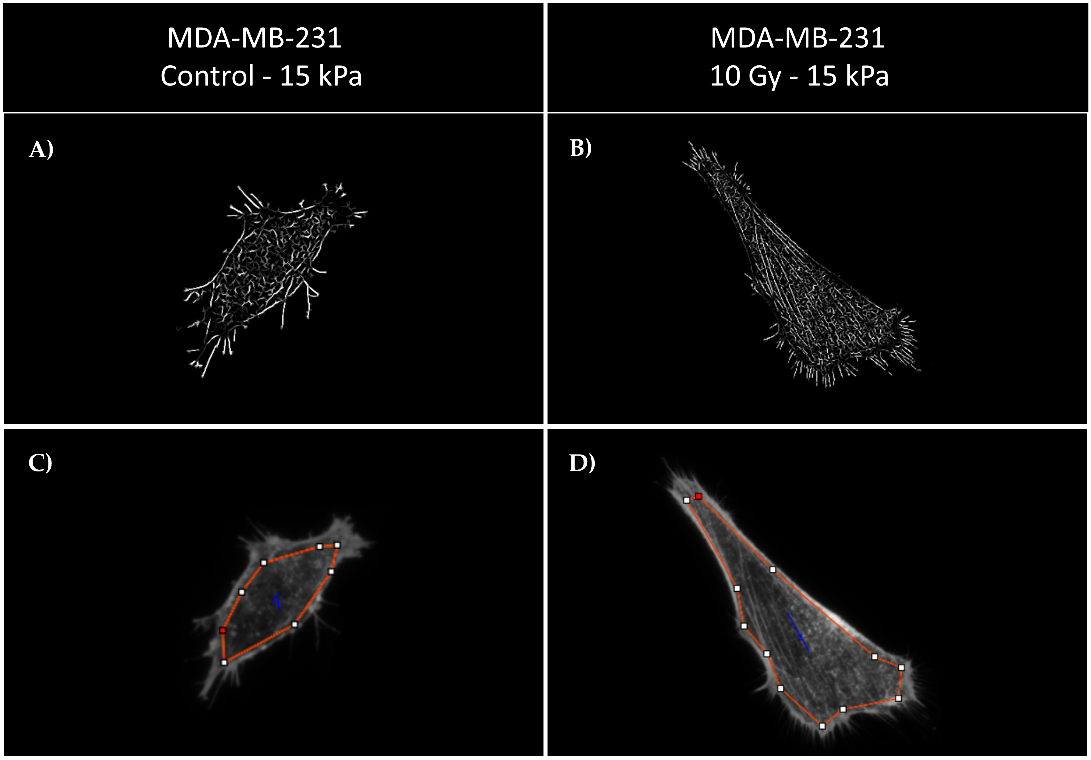
**

**Supplementary Figure 2.** (A,B) Representative images of cytoskeletal F-actin’s filaments reconstruction obtained by the MatLab^TM^ routine applied to the CSK projections on the adhesion’s plane: “Automated segmentation and quantification of actin stress fibres undergoing experimentally induced changes”. Here we reported only two reference conditions for adenocarcinoma cells (MDA-MB-231) cultured on 15 kPa, not irradiated (control)  (A) and exposed to 10 Gy (B). (C,D) Representative images of the same cells examined at the adhesion’s plane level applying the FibrilTool plug-in in Fiji Image-J: in both cases the polygonal selection (red) defines the ROI into which the level of cytoskeletal stress fibers’ anisotropy is evaluated.

*
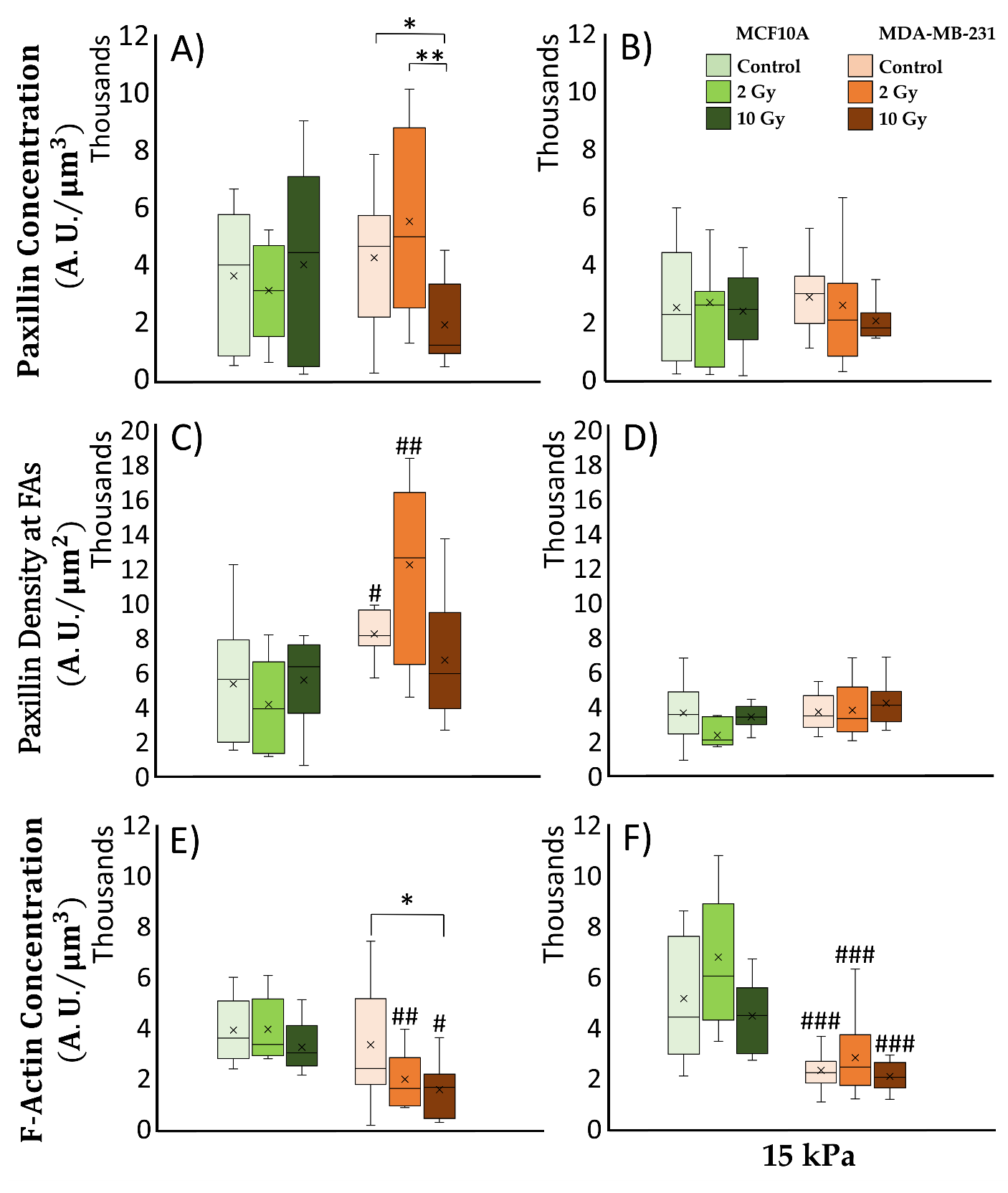
*

**Supplementary Figure 3.** Differential mechanical response to radiation of non-tumorigenic (MCF10A) and malignant cells (MDA-MB-231) evaluated in terms of paxillin cytoplasmatic concentration (A,B), paxillin density at FAs C,D) and cytoskeletal F-actin concentration (E,F) 72 h after RT. The analysis was performed for two stiffness of the ECM equal to 0.5 (A,C,E) and 15 kPa (B,D,F). Cytoplasmatic paxillin and cytoskeletal F-actin concentrations were evaluated by normalizing Integrated Density (from main text [Figure 2](https://communitystudentiunina.sharepoint.com/:w:/s/Valeria-Giuseppe-Rocco/ETjlMMs9IRRItj8J3XMMT6wB1uUR1XbhPErJHBe041wnpA) A,B and [Figure 3](https://communitystudentiunina.sharepoint.com/sites/Valeria-Giuseppe-Rocco/Shared%20Documents/General/Lavori/FrascoReale%202.0/Frontiers/Versione%20revisionata%20da%20sottomettere/1408789_Manuscript.DOCX) A,B respectively) on the single cell reconstructed total volume (from main text [Figure 5](https://communitystudentiunina.sharepoint.com/:w:/s/Valeria-Giuseppe-Rocco/ETjlMMs9IRRItj8J3XMMT6wB1uUR1XbhPErJHBe041wnpA), [Figure 6](https://communitystudentiunina.sharepoint.com/:w:/s/Valeria-Giuseppe-Rocco/ETjlMMs9IRRItj8J3XMMT6wB1uUR1XbhPErJHBe041wnpA) A,B). Similarly, paxillin density at FAs was obtained by normalizing paxillin Integrated Density (from main text [Figure 2](https://communitystudentiunina.sharepoint.com/:w:/s/Valeria-Giuseppe-Rocco/ETjlMMs9IRRItj8J3XMMT6wB1uUR1XbhPErJHBe041wnpA), C,D) on cellular spreading area (from **Supplementary Figure 1**).

## Statistical Analysis

Results for each experimental condition were presented using boxplots. The boxes, delineated by the first and third quartiles, encapsulate the median, with segments representing minimum and maximum values. The normality of data was assessed via the Shapiro–Wilk test (p < 0.05 indicating non-normal distribution). Statistical comparisons were conducted using Student’s unpaired t-test for normally distributed data, while non-normally distributed data underwent the nonparametric Kruskal–Wallis test. Statistical significance was assigned to differences with p-values < 0.05. Additional details are provided in the following Tables:

Supplementary Table 1. Statistical analysis on paxillin expression.

| **Paxillin Expression** | | | **MCF10A** | | | | | | **MDA-MB-231** | | | | | |
| --- | --- | --- | --- | --- | --- | --- | --- | --- | --- | --- | --- | --- | --- | --- |
|  |  |  | **Control** | | **2 Gy** | | **10 Gy** | | **Control** | | **2 Gy** | | **10 Gy** | |
|  |  |  | **0.5 kPa** | **15 kPa** | **0.5 kPa** | **15 kPa** | **0.5 kPa** | **15 kPa** | **0.5 kPa** | **15 kPa** | **0.5 kPa** | **15 kPa** | **0.5 kPa** | **15 kPa** |
| **MCF10A** | **Control** | **0.5 kPa** |  | 0.1342 | 0.5679 | 0.1525 | 0.9334 | 0.0356 | 0.8825 | 0.6489 | 0.0004 | 0.0507 | 0.0543 | 0.0097 |
|  |  | **15 kPa** |  |  | 0.0473 | 0.9185 | 0.1138 | 0.5457 | 0.0998 | 0.2450 | 0.0357 | 0.6481 | 0.6409 | 0.2598 |
|  | **2 Gy** | **0.5 kPa** |  |  |  | 0.0538 | 0.6224 | 0.0107 | 0.6659 | 0.2999 | 0.0001 | 0.0158 | 0.0174 | 0.0027 |
|  |  | **15 kPa** |  |  |  |  | 0.1294 | 0.4711 | 0.1136 | 0.2785 | 0.0247 | 0.5691 | 0.5644 | 0.2108 |
|  | **10 Gy** | **0.5 kPa** |  |  |  |  |  | 0.0288 | 0.9487 | 0.5854 | 0.0003 | 0.0416 | 0.0449 | 0.0077 |
|  |  | **15 kPa** |  |  |  |  |  |  | 0.0245 | 0.0695 | 0.1305 | 0.8825 | 0.9032 | 0.5900 |
| **MDA-MB-231** | **Control** | **0.5 kPa** |  |  |  |  |  |  |  | 0.5386 | 0.0002 | 0.0356 | 0.0386 | 0.0064 |
|  |  | **15 kPa** |  |  |  |  |  |  |  |  | 0.0007 | 0.0978 | 0.1035 | 0.0194 |
|  | **2 Gy** | **0.5 kPa** |  |  |  |  |  |  |  |  |  | 0.0977 | 0.1113 | 0.3428 |
|  |  | **15 kPa** |  |  |  |  |  |  |  |  |  |  | 0.9823 | 0.4948 |
|  | **10 Gy** | **0.5 kPa** |  |  |  |  |  |  |  |  |  |  |  | 0.5198 |
| **Paxillin Concentration** | | | **MCF10A** | | | | | | **MDA-MB-231** | | | | | |
|  |  |  | **Control** | | **2 Gy** | | **10 Gy** | | **Control** | | **2 Gy** | | **10 Gy** | |
|  |  |  | **0.5 kPa** | **15 kPa** | **0.5 kPa** | **15 kPa** | **0.5 kPa** | **15 kPa** | **0.5 kPa** | **15 kPa** | **0.5 kPa** | **15 kPa** | **0.5 kPa** | **15 kPa** |
| **MCF10A** | **Control** | **0.5 kPa** |  | 0.2420 | 0.7615 | 0.3221 | 0.8702 | 0.2527 | 0.6240 | 0.6630 | 0.2864 | 0.1739 | 0.0793 | 0.1392 |
|  |  | **15 kPa** |  |  | 0.4239 | 0.8357 | 0.3141 | 0.9791 | 0.0969 | 0.4317 | 0.0275 | 0.8496 | 0.5381 | 0.7338 |
|  | **2 Gy** | **0.5 kPa** |  |  |  | 0.5344 | 0.8812 | 0.4383 | 0.4438 | 0.9256 | 0.1921 | 0.3279 | 0.1729 | 0.2704 |
|  |  | **15 kPa** |  |  |  |  | 0.4105 | 0.8566 | 0.1357 | 0.5556 | 0.0401 | 0.6881 | 0.4059 | 0.5829 |
|  | **10 Gy** | **0.5 kPa** |  |  |  |  |  | 0.3268 | 0.5133 | 0.7909 | 0.2205 | 0.2316 | 0.1106 | 0.1869 |
|  |  | **15 kPa** |  |  |  |  |  |  | 0.1022 | 0.4479 | 0.0293 | 0.8292 | 0.5214 |  |
| **MDA-MB-231** | **Control** | **0.5 kPa** |  |  |  |  |  |  |  | 0.3432 | 0.5559 | 0.0643 | 0.0256 | 0.0505 |
|  |  | **15 kPa** |  |  |  |  |  |  |  |  | 0.1250 | 0.3250 | 0.1600 | 0.2637 |
|  | **2 Gy** | **0.5 kPa** |  |  |  |  |  |  |  |  |  | 0.0169 | 0.0060 | 0.0131 |
|  |  | **15 kPa** |  |  |  |  |  |  |  |  |  |  | 0.6663 | 0.8764 |
|  | **10 Gy** | **0.5 kPa** |  |  |  |  |  |  |  |  |  |  |  | 0.7881 |
| **Paxillin Expression at FAs** | | | **MCF10A** | | | | | | **MDA-MB-231** | | | | | |
|  |  |  | **Control** | | **2 Gy** | | **10 Gy** | | **Control** | | **2 Gy** | | **10 Gy** | |
|  |  |  | **0.5 kPa** | **15 kPa** | **0.5 kPa** | **15 kPa** | **0.5 kPa** | **15 kPa** | **0.5 kPa** | **15 kPa** | **0.5 kPa** | **15 kPa** | **0.5 kPa** | **15 kPa** |
| **MCF10A** | **Control** | **0.5 kPa** |  | 0.0018 | 0.5845 | 0.1400 | 0.7999 | 0.0000 | 0.6214 | 0.2105 | 0.0002 | 0.0032 | 0.0608 | 0.0266 |
|  |  | **15 kPa** |  |  | 0.0005 | 0.1473 | 0.0014 | 0.3506 | 0.0129 | 0.0532 | 0.6209 | 0.9338 | 0.2397 | 0.5387 |
|  | **2 Gy** | **0.5 kPa** |  |  |  | 0.0566 | 0.7809 | 0.0000 | 0.3235 | 0.0841 | 0.0000 | 0.0010 | 0.0216 | 0.0093 |
|  |  | **15 kPa** |  |  |  |  | 0.1015 | 0.0187 | 0.3409 | 0.7344 | 0.0494 | 0.1531 | 0.7540 | 0.4525 |
|  | **10 Gy** | **0.5 kPa** |  |  |  |  |  | 0.0000 | 0.4783 | 0.1515 | 0.0002 | 0.0024 | 0.0435 | 0.0191 |
|  |  | **15 kPa** |  |  |  |  |  |  | 0.0006 | 0.0035 | 0.6413 | 0.4348 | 0.0349 | 0.1392 |
| **MDA-MB-231** | **Control** | **0.5 kPa** |  |  |  |  |  |  |  | 0.4922 | 0.0023 | 0.0171 | 0.1901 | 0.0917 |
|  |  | **15 kPa** |  |  |  |  |  |  |  |  | 0.0118 | 0.0625 | 0.4875 | 0.2572 |
|  | **2 Gy** | **0.5 kPa** |  |  |  |  |  |  |  |  |  | 0.7108 | 0.0883 | 0.2775 |
|  |  | **15 kPa** |  |  |  |  |  |  |  |  |  |  | 0.2414 | 0.5138 |
|  | **10 Gy** | **0.5 kPa** |  |  |  |  |  |  |  |  |  |  |  | 0.6358 |
| **Paxillin Density at FAs** | | | **MCF10A** | | | | | | **MDA-MB-231** | | | | | |
|  |  |  | **Control** | | **2 Gy** | | **10 Gy** | | **Control** | | **2 Gy** | | **10 Gy** | |
|  |  |  | **0.5 kPa** | **15 kPa** | **0.5 kPa** | **15 kPa** | **0.5 kPa** | **15 kPa** | **0.5 kPa** | **15 kPa** | **0.5 kPa** | **15 kPa** | **0.5 kPa** | **15 kPa** |
| **MCF10A** | **Control** | **0.5 kPa** |  | 0.3430 | 0.5206 | 0.0246 | 0.5547 | 0.2545 | 0.0170 | 0.4905 | 0.0058 | 0.4301 | 0.3211 | 0.7484 |
|  |  | **15 kPa** |  |  | 0.7717 | 0.2035 | 0.1443 | 0.8561 | 0.0016 | 0.7843 | 0.0004 | 0.9375 | 0.0656 | 0.5988 |
|  | **2 Gy** | **0.5 kPa** |  |  |  | 0.1207 | 0.2421 | 0.6372 | 0.0040 | 0.9801 | 0.0012 | 0.8491 | 0.1210 | 0.7968 |
|  |  | **15 kPa** |  |  |  |  | 0.0073 | 0.2729 | 0.0000 | 0.1165 | 0.0000 | 0.2080 | 0.0023 | 0.0903 |
|  | **10 Gy** | **0.5 kPa** |  |  |  |  |  | 0.1007 | 0.0882 | 0.2192 | 0.0422 | 0.2029 | 0.7033 | 0.4090 |
|  |  | **15 kPa** |  |  |  |  |  |  | 0.0008 | 0.6453 | 0.0002 | 0.8055 | 0.0432 | 0.4877 |
| **MDA-MB-231** | **Control** | **0.5 kPa** |  |  |  |  |  |  |  | 0.0029 | 0.7819 | 0.0043 | 0.1855 | 0.0162 |
|  |  | **15 kPa** |  |  |  |  |  |  |  |  | 0.0008 | 0.8634 | 0.1051 | 0.7743 |
|  | **2 Gy** | **0.5 kPa** |  |  |  |  |  |  |  |  |  | 0.0015 | 0.1012 | 0.0066 |
|  |  | **15 kPa** |  |  |  |  |  |  |  |  |  |  | 0.1040 | 0.6753 |
|  | **10 Gy** | **0.5 kPa** |  |  |  |  |  |  |  |  |  |  |  | 0.2387 |

Supplementary Table 2. Statistical analysis on F-actin’s expression and fibers length (median/total) and anisotropy

| **F-Actin Expression** | | | | **MCF10A** | | | | | | | | | | | | | **MDA-MB-231** | | | | | | | | | | |
| --- | --- | --- | --- | --- | --- | --- | --- | --- | --- | --- | --- | --- | --- | --- | --- | --- | --- | --- | --- | --- | --- | --- | --- | --- | --- | --- | --- |
|  |  |  |  | **Control** | | | | **2 Gy** | | | | | | **10 Gy** | | | **Control** | | | | **2 Gy** | | | | **10 Gy** | | |
|  |  |  |  | **0.5 kPa** | | **15 kPa** | | **0.5 kPa** | | | **15 kPa** | | | **0.5 kPa** | | **15 kPa** | **0.5 kPa** | | **15 kPa** | | **0.5 kPa** | | | **15 kPa** | **0.5 kPa** | | **15 kPa** |
| **MCF10A** | **Control** | | **0.5 kPa** |  | | 0.0003 | | 0.7665 | | | 0.0000 | | | 0.3647 | | 0.0001 | 0.2859 | | 0.6967 | | 0.3833 | | | 0.0087 | 0.2389 | | 0.0393 |
|  |  |  | **15 kPa** |  | |  | | 0.0002 | | | 0.6864 | | | 0.0000 | | 0.8420 | 0.0000 | | 0.0000 | | 0.0083 | | | 0.3254 | 0.0197 | | 0.1472 |
|  | **2 Gy** | | **0.5 kPa** |  | |  | |  | | | 0.0000 | | | 0.5771 | | 0.0001 | 0.4783 | | 0.9630 | | 0.2652 | | | 0.0056 | 0.1605 | | 0.0252 |
|  |  |  | **15 kPa** |  | |  | |  | | |  | | | 0.0000 | | 0.8417 | 0.0000 | | 0.0000 | | 0.0020 | | | 0.1584 | 0.0055 | | 0.0609 |
|  | **10 Gy** | | **0.5 kPa** |  | |  | |  | | |  | | |  | | 0.0000 | 0.8723 | | 0.5557 | | 0.0794 | | | 0.0004 | 0.0394 | | 0.0033 |
|  |  |  | **15 kPa** |  | |  | |  | | |  | | |  | |  | 0.0000 | | 0.0000 | | 0.0046 | | | 0.2369 | 0.0115 | | 0.1003 |
| **MDA-MB-231** | **Control** | | **0.5 kPa** |  | |  | |  | | |  | | |  | |  |  | | 0.4456 | | 0.0561 | | | 0.0002 | 0.0267 | | 0.0019 |
|  |  |  | **15 kPa** |  | |  | |  | | |  | | |  | |  |  | |  | | 0.1884 | | | 0.0013 | 0.1001 | | 0.0095 |
|  | **2 Gy** | | **0.5 kPa** |  | |  | |  | | |  | | |  | |  |  | |  | |  | | | 0.0928 | 0.7656 | | 0.2466 |
|  |  |  | **15 kPa** |  | |  | |  | | |  | | |  | |  |  | |  | |  | | |  | 0.1691 | | 0.6226 |
|  | **10 Gy** | | **0.5 kPa** |  | |  | |  | | |  | | |  | |  |  | |  | |  | | |  |  | | 0.3895 |
| **F-Actin Concentration** | | | | **MCF10A** | | | | | | | | | | | | | **MDA-MB-231** | | | | | | | | | | |
|  |  |  |  | **Control** | | | | **2 Gy** | | | | | | **10 Gy** | | | **Control** | | | | **2 Gy** | | | | **10 Gy** | | |
|  |  |  |  | **0.5 kPa** | | **15 kPa** | | **0.5 kPa** | | | **15 kPa** | | | **0.5 kPa** | | **15 kPa** | **0.5 kPa** | | **15 kPa** | | **0.5 kPa** | | | **15 kPa** | **0.5 kPa** | | **15 kPa** |
| **MCF10A** | **Control** | | **0.5 kPa** |  | | 0.4598 | | 0.8939 | | | 0.0698 | | | 0.4254 | | 0.5329 | 0.1812 | | 0.0109 | | 0.0065 | | | 0.0897 | 0.0011 | | 0.0058 |
|  |  |  | **15 kPa** |  | |  | | 0.5730 | | | 0.2906 | | | 0.1245 | | 0.9079 | 0.0379 | | 0.0008 | | 0.0006 | | | 0.0148 | 0.0001 | | 0.0005 |
|  | **2 Gy** | | **0.5 kPa** |  | |  | |  | | | 0.1166 | | | 0.3762 | | 0.6494 | 0.1633 | | 0.0117 | | 0.0069 | | | 0.0830 | 0.0012 | | 0.0061 |
|  |  |  | **15 kPa** |  | |  | |  | | |  | | | 0.0086 | | 0.2399 | 0.0015 | | 0.0000 | | 0.0000 | | | 0.0004 | 0.0000 | | 0.0000 |
|  | **10 Gy** | | **0.5 kPa** |  | |  | |  | | |  | | |  | | 0.1554 | 0.5892 | | 0.0921 | | 0.0519 | | | 0.3681 | 0.0124 | | 0.0471 |
|  |  |  | **15 kPa** |  | |  | |  | | |  | | |  | |  | 0.0499 | | 0.0013 | | 0.0009 | | | 0.0203 | 0.0001 | | 0.0008 |
| **MDA-MB-231** | **Control** | | **0.5 kPa** |  | |  | |  | | |  | | |  | |  |  | | 0.2709 | | 0.1561 | | | 0.7189 | 0.0483 | | 0.1443 |
|  |  |  | **15 kPa** |  | |  | |  | | |  | | |  | |  |  | |  | | 0.6468 | | | 0.4764 | 0.2908 | | 0.6149 |
|  | **2 Gy** | | **0.5 kPa** |  | |  | |  | | |  | | |  | |  |  | |  | |  | | | 0.2856 | 0.5878 | | 0.9676 |
|  |  |  | **15 kPa** |  | |  | |  | | |  | | |  | |  |  | |  | |  | | |  | 0.1044 | | 0.2672 |
|  | **10 Gy** | | **0.5 kPa** |  | |  | |  | | |  | | |  | |  |  | |  | |  | | |  |  | | 0.6161 |
| **F-Actin Median Fiber Length** | | | | **MCF10A** | | | | | | | | | | | | | **MDA-MB-231** | | | | | | | | | | |
|  |  |  |  | **Control** | | | | **2 Gy** | | | | | | **10 Gy** | | | **Control** | | | | **2 Gy** | | | | **10 Gy** | | |
|  |  |  |  | **0.5 kPa** | | **15 kPa** | | **0.5 kPa** | | | **15 kPa** | | | **0.5 kPa** | | **15 kPa** | **0.5 kPa** | | **15 kPa** | | **0.5 kPa** | | | **15 kPa** | **0.5 kPa** | | **15 kPa** |
| **MCF10A** | **Control** | | **0.5 kPa** |  | | 0.3385 | | 0.3000 | | | 0.9715 | | | 0.5042 | | 0.0667 | 0.2463 | | 0.5132 | | 0.0145 | | | 0.9543 | 0.3747 | | 0.8215 |
|  |  |  | **15 kPa** |  | |  | | 0.0568 | | | 0.3482 | | | 0.1042 | | 0.3668 | 0.0365 | | 0.7160 | | 0.0007 | | | 0.3227 | 0.0734 | | 0.4802 |
|  | **2 Gy** | | **0.5 kPa** |  | |  | |  | | | 0.3400 | | | 0.6670 | | 0.0072 | 0.9652 | | 0.0937 | | 0.2373 | | | 0.3365 | 0.8625 | | 0.2228 |
|  |  |  | **15 kPa** |  | |  | |  | | |  | | | 0.5525 | | 0.0769 | 0.2885 | | 0.5159 | | 0.0233 | | | 0.9846 | 0.4189 | | 0.8040 |
|  | **10 Gy** | | **0.5 kPa** |  | |  | |  | | |  | | |  | | 0.0130 | 0.6105 | | 0.1726 | | 0.0757 | | | 0.5534 | 0.7964 | | 0.3812 |
|  |  |  | **15 kPa** |  | |  | |  | | |  | | |  | |  | 0.0035 | | 0.1905 | | 0.0000 | | | 0.0653 | 0.0093 | | 0.1170 |
| **MDA-MB-231** | **Control** | | **0.5 kPa** |  | |  | |  | | |  | | |  | |  |  | | 0.0625 | | 0.2226 | | | 0.2828 | 0.8183 | | 0.1770 |
|  |  |  | **15 kPa** |  | |  | |  | | |  | | |  | |  |  | |  | | 0.0011 | | | 0.4871 | 0.1214 | | 0.6928 |
|  | **2 Gy** | | **0.5 kPa** |  | |  | |  | | |  | | |  | |  |  | |  | |  | | | 0.0202 | 0.1565 | | 0.0092 |
|  |  |  | **15 kPa** |  | |  | |  | | |  | | |  | |  |  | |  | |  | | |  | 0.4166 | | 0.7827 |
|  | **10 Gy** | | **0.5 kPa** |  | |  | |  | | |  | | |  | |  |  | |  | |  | | |  |  | | 0.2802 |
| **F-Actin Total Fiber Length** | | | | **MCF10A** | | | | | | | | | | | | | **MDA-MB-231** | | | | | | | | | | |
|  |  |  |  | **Control** | | | **2 Gy** | | | | | | **10 Gy** | | | | **Control** | | | | **2 Gy** | | | | **10 Gy** | | |
|  |  |  |  | **0.5 kPa** | **15 kPa** | | **0.5 kPa** | | | **15 kPa** | | | **0.5 kPa** | | **15 kPa** | | **0.5 kPa** | **15 kPa** | | | **0.5 kPa** | | **15 kPa** | | **0.5 kPa** | **15 kPa** | |
| **MCF10A** | **Control** | **0.5 kPa** | |  | 5.3E-06 | | 7.0E-01 | | | 2.4E-04 | | | 8.3E-01 | | 9.9E-07 | | 9.2E-01 | 8.1E-02 | | | 1.8E-01 | | 1.5E-05 | | 3.3E-02 | 2.0E-05 | |
|  |  | **15 kPa** | |  |  | | 6.3E-06 | | | 5.3E-01 | | | 1.8E-06 | | 7.3E-01 | | 1.5E-05 | 2.0E-03 | | | 1.3E-03 | | 9.2E-01 | | 3.1E-02 | 8.7E-01 | |
|  | **2 Gy** | **0.5 kPa** | |  |  | |  | | | 1.9E-04 | | | 8.5E-01 | | 1.4E-06 | | 6.4E-01 | 4.9E-02 | | | 1.1E-01 | | 1.5E-05 | | 2.0E-02 | 2.0E-05 | |
|  |  | **15 kPa** | |  |  | |  | | |  | | | 1.1E-04 | | 3.5E-01 | | 5.0E-04 | 2.5E-02 | | | 1.6E-02 | | 6.1E-01 | | 1.5E-01 | 6.5E-01 | |
|  | **10 Gy** | **0.5 kPa** | |  |  | |  | | |  | | |  | | 3.2E-07 | | 7.5E-01 | 4.8E-02 | | | 1.2E-01 | | 5.5E-06 | | 1.9E-02 | 7.5E-06 | |
|  |  | **15 kPa** | |  |  | |  | | |  | | |  | |  | | 3.2E-06 | 5.5E-04 | | | 3.8E-04 | | 6.7E-01 | | 1.3E-02 | 6.2E-01 | |
| **MDA-MB-231** | **Control** | **0.5 kPa** | |  |  | |  | | |  | | |  | |  | |  | 1.1E-01 | | | 2.3E-01 | | 3.8E-05 | | 4.7E-02 | 5.0E-05 | |
|  |  | **15 kPa** | |  |  | |  | | |  | | |  | |  | |  |  | | | 7.5E-01 | | 3.8E-03 | | 5.4E-01 | 4.7E-03 | |
|  | **2 Gy** | **0.5 kPa** | |  |  | |  | | |  | | |  | |  | |  |  | | |  | | 2.4E-03 | | 3.8E-01 | 3.0E-03 | |
|  |  | **15 kPa** | |  |  | |  | | |  | | |  | |  | |  |  | | |  | |  | | 4.4E-02 | 9.5E-01 | |
|  | **10 Gy** | **0.5 kPa** | |  |  | |  | | |  | | |  | |  | |  |  | | |  | |  | |  | 5.1E-02 | |
| **CSK Anisotropy** | | | | **MCF10A** | | | | | | | | | | | | | **MDA-MB-231** | | | | | | | | | | |
|  |  |  |  | **Control** | | | | | **2 Gy** | | | | | **10 Gy** | | | **Control** | | | | | **2 Gy** | | | **10 Gy** | | |
|  |  |  |  | **0.5 kPa** | | **15 kPa** | | | **0.5 kPa** | | | **15 kPa** | | **0.5 kPa** | | **15 kPa** | **0.5 kPa** | | | **15 kPa** | | **0.5 kPa** | | **15 kPa** | **0.5 kPa** | | **15 kPa** |
| **MCF10A** | **Control** | | **0.5 kPa** |  | | 2.1E-06 | | | 0.9597 | | | 0.0004 | | 0.5778 | | 3.6E-06 | 0.4472 | | | 0.0479 | | 0.8578 | | 0.0255 | 0.4455 | | 0.0185 |
|  |  |  | **15 kPa** |  | |  | | | 6.2E-06 | | | 0.4520 | | 2.9E-05 | | 0.9145 | 0.0001 | | | 0.0017 | | 1.6E-06 | | 0.0121 | 7.5E-08 | | 0.0239 |
|  | **2 Gy** | | **0.5 kPa** |  | |  | | |  | | | 0.0006 | | 0.5650 | | 9.9E-06 | 0.4424 | | | 0.0572 | | 0.9045 | | 0.0311 | 0.5015 | | 0.0228 |
|  |  |  | **15 kPa** |  | |  | | |  | | |  | | 0.0023 | | 0.5127 | 0.0054 | | | 0.0443 | | 0.0003 | | 0.1275 | 3.1E-05 | | 0.1854 |
|  | **10 Gy** | | **0.5 kPa** |  | |  | | |  | | |  | |  | | 4.6E-05 | 0.8272 | | | 0.1686 | | 0.4709 | | 0.0936 | 0.1920 | | 0.0697 |
|  |  |  | **15 kPa** |  | |  | | |  | | |  | |  | |  | 0.0002 | | | 0.0025 | | 2.7E-06 | | 0.0164 | 1.3E-07 | | 0.0312 |
| **MDA-MB-231** | **Control** | | **0.5 kPa** |  | |  | | |  | | |  | |  | |  |  | | | 0.2715 | | 0.3599 | | 0.1575 | 0.1377 | | 0.1199 |
|  |  |  | **15 kPa** |  | |  | | |  | | |  | |  | |  |  | | |  | | 0.0349 | | 0.6642 | 0.0062 | | 0.5376 |
|  | **2 Gy** | | **0.5 kPa** |  | |  | | |  | | |  | |  | |  |  | | |  | |  | | 0.0186 | 0.5693 | | 0.0135 |
|  |  |  | **15 kPa** |  | |  | | |  | | |  | |  | |  |  | | |  | |  | |  | 0.0033 | | 0.8555 |
|  | **10 Gy** | | **0.5 kPa** |  | |  | | |  | | |  | |  | |  |  | | |  | |  | |  |  | | 0.0024 |

Supplementary Table 3. Statistical analysis on cellular Young’s modulus.

| **Cellular Young’s Modulus** | | | **MCF10A** | | | | | | **MDA-MB-231** | | | | | |
| --- | --- | --- | --- | --- | --- | --- | --- | --- | --- | --- | --- | --- | --- | --- |
|  |  |  | **Control** | | **2 Gy** | | **10 Gy** | | **Control** | | **2 Gy** | | **10 Gy** | |
|  |  |  | **0.5 kPa** | **15 kPa** | **0.5 kPa** | **15 kPa** | **0.5 kPa** | **15 kPa** | **0.5 kPa** | **15 kPa** | **0.5 kPa** | **15 kPa** | **0.5 kPa** | **15 kPa** |
| **MCF10A** | **Control** | **0.5 kPa** |  | 0.0E+00 | 9.7E-02 | 0.0E+00 | 2.4E-01 | 0.0E+00 | 2.2E-03 | 2.3E-07 | 1.7E-01 | 0.0E+00 | 9.2E-01 | 0.0E+00 |
|  |  | **15 kPa** |  |  | 1.1E-14 | 2.4E-01 | 0.0E+00 | 5.4E-01 | 0.0E+00 | 2.6E-05 | 0.0E+00 | 7.9E-01 | 0.0E+00 | 1.8E-01 |
|  | **2 Gy** | **0.5 kPa** |  |  |  | 0.0E+00 | 5.5E-01 | 0.0E+00 | 9.6E-07 | 5.2E-04 | 2.1E-03 | 4.4E-16 | 7.0E-02 | 0.0E+00 |
|  |  | **15 kPa** |  |  |  |  | 0.0E+00 | 5.6E-01 | 0.0E+00 | 4.0E-08 | 0.0E+00 | 1.2E-01 | 0.0E+00 | 8.9E-01 |
|  | **10 Gy** | **0.5 kPa** |  |  |  |  |  | 0.0E+00 | 3.7E-06 | 1.7E-05 | 7.6E-03 | 0.0E+00 | 2.1E-01 | 0.0E+00 |
|  |  | **15 kPa** |  |  |  |  |  |  | 0.0E+00 | 7.9E-07 | 0.0E+00 | 3.4E-01 | 0.0E+00 | 4.7E-01 |
| **MDA-MB-231** | **Control** | **0.5 kPa** |  |  |  |  |  |  |  | 0.0E+00 | 1.1E-01 | 0.0E+00 | 1.5E-04 | 0.0E+00 |
|  |  | **15 kPa** |  |  |  |  |  |  |  |  | 2.3E-11 | 1.5E-05 | 5.0E-09 | 1.1E-08 |
|  | **2 Gy** | **0.5 kPa** |  |  |  |  |  |  |  |  |  | 0.0E+00 | 8.9E-02 | 0.0E+00 |
|  |  | **15 kPa** |  |  |  |  |  |  |  |  |  |  | 0.0E+00 | 7.8E-02 |
|  | **10 Gy** | **0.5 kPa** |  |  |  |  |  |  |  |  |  |  |  | 0.0E+00 |
|  |  | **15 kPa** |  |  |  |  |  |  |  |  |  |  |  |  |

Supplementary Table 4. Statistical analysis on cellular and nuclear volumes.

| **Cellular Volume** | | | **MCF10A** | | | | | | | | | | | **MDA-MB-231** | | | | | | | |
| --- | --- | --- | --- | --- | --- | --- | --- | --- | --- | --- | --- | --- | --- | --- | --- | --- | --- | --- | --- | --- | --- |
|  |  |  | **Control** | | | | **2 Gy** | | | | **10 Gy** | | | **Control** | | **2 Gy** | | | **10 Gy** | | |
|  |  |  | **0.5 kPa** | | **15 kPa** | | **0.5 kPa** | | **15 kPa** | | **0.5 kPa** | | **15 kPa** | **0.5 kPa** | **15 kPa** | **0.5 kPa** | **15 kPa** | | **0.5 kPa** | | **15 kPa** |
| **MCF10A** | **Control** | **0.5 kPa** |  | | 0.0014 | | 0.6975 | | 0.0198 | | 0.5674 | | 0.0001 | 0.9523 | 0.3142 | 0.0014 | 0.0000 | | 0.0000 | | 0.0000 |
|  |  | **15 kPa** |  | |  | | 0.0006 | | 0.4895 | | 0.0002 | | 0.4525 | 0.0017 | 0.0141 | 0.8582 | 0.3623 | | 0.2444 | | 0.1767 |
|  | **2 Gy** | **0.5 kPa** |  | |  | |  | | 0.0099 | | 0.8804 | | 0.0000 | 0.6563 | 0.1750 | 0.0007 | 0.0000 | | 0.0000 | | 0.0000 |
|  |  | **15 kPa** |  | |  | |  | |  | | 0.0041 | | 0.1617 | 0.0230 | 0.1202 | 0.4093 | 0.1212 | | 0.0758 | | 0.0509 |
|  | **10 Gy** | **0.5 kPa** |  | |  | |  | |  | |  | | 0.0000 | 0.5276 | 0.1044 | 0.0002 | 0.0000 | | 0.0000 | | 0.0000 |
|  |  | **15 kPa** |  | |  | |  | |  | |  | |  | 0.0001 | 0.0011 | 0.5963 | 0.8732 | | 0.6651 | | 0.5354 |
| **MDA-MB-231** | **Control** | **0.5 kPa** |  | |  | |  | |  | |  | |  |  | 0.3463 | 0.0017 | 0.0000 | | 0.0000 | | 0.0000 |
|  |  | **15 kPa** |  | |  | |  | |  | |  | |  |  |  | 0.0130 | 0.0006 | | 0.0003 | | 0.0001 |
|  | **2 Gy** | **0.5 kPa** |  | |  | |  | |  | |  | |  |  |  |  | 0.4964 | | 0.3542 | | 0.2699 |
|  |  | **15 kPa** |  | |  | |  | |  | |  | |  |  |  |  |  | | 0.7813 | | 0.6423 |
|  | **10 Gy** | **0.5 kPa** |  | |  | |  | |  | |  | |  |  |  |  |  | |  | | 0.8554 |
| **Nuclear Volume** | | | **MCF10A** | | | | | | | | | | | **MDA-MB-231** | | | | | | | |
|  |  |  | **Control** | | | **2 Gy** | | | | **10 Gy** | | | | **Control** | | **2 Gy** | | **10 Gy** | | | |
|  |  |  | **0.5 kPa** | **15 kPa** | | **0.5 kPa** | | **15 kPa** | | **0.5 kPa** | | **15 kPa** | | **0.5 kPa** | **15 kPa** | **0.5 kPa** | **15 kPa** | **0.5 kPa** | | **15 kPa** | |
| **MCF10A** | **Control** | **0.5 kPa** |  | 0.1212 | | 0.7579 | | 0.0306 | | 0.1202 | | 0.0013 | | 0.0435 | 5.2E-07 | 0.1704 | 2.2E-06 | 0.0291 | | 2.9E-09 | |
|  |  | **15 kPa** |  |  | | 0.0327 | | 0.3457 | | 0.8752 | | 0.0449 | | 0.3909 | 4.1E-05 | 0.9938 | 0.0002 | 0.3637 | | 4.1E-07 | |
|  | **2 Gy** | **0.5 kPa** |  |  | |  | | 0.0078 | | 0.0399 | | 0.0001 | | 0.0139 | 8.3E-09 | 0.0684 | 2.3E-08 | 0.0065 | | 3.3E-12 | |
|  |  | **15 kPa** |  |  | |  | |  | | 0.4596 | | 0.4326 | | 0.9852 | 0.0057 | 0.4025 | 0.0227 | 0.9407 | | 0.0008 | |
|  | **10 Gy** | **0.5 kPa** |  |  | |  | |  | |  | | 0.0945 | | 0.4987 | 0.0002 | 0.8962 | 0.0011 | 0.4875 | | 8.2E-06 | |
|  |  | **15 kPa** |  |  | |  | |  | |  | |  | | 0.4487 | 0.0256 | 0.0816 | 0.0953 | 0.3656 | | 0.0042 | |
| **MDA-MB-231** | **Control** | **0.5 kPa** |  |  | |  | |  | |  | |  | |  | 0.0082 | 0.4403 | 0.0302 | 0.9593 | | 0.0014 | |
|  |  | **15 kPa** |  |  | |  | |  | |  | |  | |  |  | 0.0002 | 0.4846 | 0.0033 | | 0.7535 | |
|  | **2 Gy** | **0.5 kPa** |  |  | |  | |  | |  | |  | |  |  |  | 0.0011 | 0.4256 | | 9.5E-06 | |
|  |  | **15 kPa** |  |  | |  | |  | |  | |  | |  |  |  |  | 0.0138 | | 0.2611 | |
|  | **10 Gy** | **0.5 kPa** |  |  | |  | |  | |  | |  | |  |  |  |  |  | | 0.0003 | |

Supplementary Table 5. Statistical analysis on cellular spreading areas.

| **Spreading Area** | | | **MCF10A** | | | | | | **MDA-MB-231** | | | | | |
| --- | --- | --- | --- | --- | --- | --- | --- | --- | --- | --- | --- | --- | --- | --- |
|  |  |  | **Control** | | **2 Gy** | | **10 Gy** | | **Control** | | **2 Gy** | | **10 Gy** | |
|  |  |  | **0.5 kPa** | **15 kPa** | **0.5 kPa** | **15 kPa** | **0.5 kPa** | **15 kPa** | **0.5 kPa** | **15 kPa** | **0.5 kPa** | **15 kPa** | **0.5 kPa** | **15 kPa** |
| **MCF10A** | **Control** | **0.5 kPa** |  | 2.6E-06 | 8.5E-01 | 2.3E-07 | 9.0E-01 | 7.2E-07 | 9.9E-01 | 2.1E-02 | 1.0E-01 | 7.4E-06 | 1.1E-02 | 3.6E-05 |
|  |  | **15 kPa** |  |  | 2.2E-05 | 7.2E-01 | 1.4E-06 | 8.0E-01 | 2.5E-06 | 5.8E-03 | 3.4E-03 | 8.3E-01 | 4.1E-02 | 6.6E-01 |
|  | **2 Gy** | **0.5 kPa** |  |  |  | 2.9E-06 | 7.6E-01 | 7.1E-06 | 8.5E-01 | 4.9E-02 | 1.7E-01 | 5.3E-05 | 2.7E-02 | 1.9E-04 |
|  |  | **15 kPa** |  |  |  |  | 1.1E-07 | 9.2E-01 | 2.2E-07 | 1.2E-03 | 8.0E-04 | 5.6E-01 | 1.5E-02 | 4.2E-01 |
|  | **10 Gy** | **0.5 kPa** |  |  |  |  |  | 3.7E-07 | 9.0E-01 | 1.4E-02 | 7.7E-02 | 4.0E-06 | 7.9E-03 | 2.1E-05 |
|  |  | **15 kPa** |  |  |  |  |  |  | 7.0E-07 | 2.4E-03 | 1.5E-03 | 6.3E-01 | 2.2E-02 | 4.9E-01 |
| **MDA-MB-231** | **Control** | **0.5 kPa** |  |  |  |  |  |  |  | 2.0E-02 | 9.9E-02 | 7.2E-06 | 1.1E-02 | 3.5E-05 |
|  |  | **15 kPa** |  |  |  |  |  |  |  |  | 6.3E-01 | 1.2E-02 | 6.3E-01 | 2.8E-02 |
|  | **2 Gy** | **0.5 kPa** |  |  |  |  |  |  |  |  |  | 6.5E-03 | 3.9E-01 | 1.5E-02 |
|  |  | **15 kPa** |  |  |  |  |  |  |  |  |  |  | 6.7E-02 | 8.2E-01 |
|  | **10 Gy** | **0.5 kPa** |  |  |  |  |  |  |  |  |  |  |  | 1.2E-01 |
